# Wenxin Keli for the Treatment of Arrhythmia—Systems Pharmacology and *In Vivo* Pharmacological Assessment

**DOI:** 10.3389/fphar.2021.704622

**Published:** 2021-08-26

**Authors:** Xiaofeng Li, Gang Tian, Liang Xu, Lili Sun, Rui Tao, Shaoqiang Zhang, Zidong Cong, Fangjun Deng, Jinhong Chen, Yang Yu, Wuxun Du, Hucheng Zhao

**Affiliations:** ^1^ Department of Cardiology, The Second Affiliated Hospital of Tianjin University of TCM, Tianjin, China; ^2^ Department of Cardiology, Teda International Cardiovascular Hospital, Tianjin, China; ^3^ School of Pharmacy, Tianjin Medical University, Tianjin, China; ^4^ Tianjin Medical College, Tianjin, China; ^5^ Department of TCM, Tianjin University of TCM, Tianjin, China; ^6^ Department of Aeronautics and Astronautics, Tsinghua University, Beijing, China

**Keywords:** Wenxin Keli, arrhythmia, active compounds, action mechanisms, systems phar macology, Ca^2+^ balance

## Abstract

This study employed a systems pharmacology approach to identify the active compounds and action mechanisms of Wenxin Keli for arrhythmia treatment. Sixty-eight components identified *in vivo* and *in vitro* by UPLC/Q-TOF-MS were considered the potential active components of Wenxin Keli. Network pharmacology further revealed 33 key targets and 75 KEGG pathways as possible pathways and targets involved in WK-mediated treatment, with the CaMKII/CNCA1C/Ca^2+^ pathway being the most significantly affected. This finding was validated using an AC-induced rat arrhythmias model. Pretreatment with Wenxin Keli reduced the malignant arrhythmias and shortened RR, PR, and the QT interval. Wenxin Keli exerted some antiarrhythmic effects by inhibiting p-CaMKII and intracellular Ca^2+^ transients and overexpressing CNCA1C. Thus, suppressing SR Ca^2+^ release and maintaining intracellular Ca^2+^ balance may be the primary mechanism of Wenxin Keli against arrhythmia. In view of the significance of CaMKII and NCX identified in this experiment, we suggest that CaMKII and NCX are essential targets for treating arrhythmias.

## Introduction

Arrhythmias are a group of conditions that cause the heart to beat irregularly (O'Rourke et al., 2016). It may remain asymptomatic or lead to other cardiovascular disorders, heart failure, stroke, and cardiac arrest. Severe arrhythmias, such as ventricular arrhythmias, have limited treatment options, causing them to be fatal (Hong et al., 2019). Numerous ongoing studies have reported treatment solutions for arrhythmias (Tian et al., 2019). Effective pharmaceutical agents with fewer side effects are also being explored. However, the progress is not satisfactory (Weiss et al., 2015; Camm, 2017). Though ion channels drug discovery has greatly evolved for nearly half a century, the drugs cause adverse reactions (Frommeyer and Eckardt, 2016), such as thyroid dysfunction, pulmonary fibrosis, and anaphylaxis (Salerno et al., 1990; Johannes et al., 2010; John et al., 2012). In recent years, ablation-guided electrophysiology and implantable cardioverter defibrillators have been widely used in arrhythmia treatment, achieving gratifying results (McKenna et al., 2019). Nonetheless, their promotion and use are limited by high cost, necessitating effective and cost-friendly medications.

Arrhythmias have a complex etiology and thus cannot be successfully treated using single-target therapies. As such, the multi-target treatment mode of traditional Chinese medicine (TCM) is handy in arrhythmia treatment. TCM is widely and frequently used in China and abroad (Wang et al., 2016; Liang et al., 2018). Wenxin Keli (WK) and Shensong Yangxin capsules are the most used TCM (Liang et al., 2019). Therefore, their antiarrhythmic mechanisms have been discovered (Luo et al., 2017; Zhang et al., 2017; Tian et al., 2018). WK is a Chinese herb extract comprised five components: *Codonopsis pilosula* (Franch.) Nannf., *Panax notoginseng* (Burkill) F.H.Chen, *Nardostachys jatamansi* (D.Don) DC., Amber, and *Polygonatum kingianum* Collett & Hemsl. It is reported to be effective in arrhythmias treatment (Burashnikov et al., 2012) and is the first TCM to be approved by the Chinese state for arrhythmias management. Numerous clinical trials postulate that WK blocks the transient outward potassium channel current (Ito), sodium current (INa), and L-type calcium current (ICaL) in rat and rabbit ventricular cardiomyocytes (Liu et al., 2009; Wang et al., 2013). It also significantly shortens APD90, thus making it incompatible with the late INa blockade, which usually produces only moderate shortening of the action potential duration (APD) (Burashnikov et al., 2012). Nevertheless, the action mechanism by which WK treats arrhythmias remains unknown because the complex chemical composition and therapeutic targets of WK challenge pharmacological investigations. As such, an effective method that can decipher the relationships between the WK and arrhythmia is needed.

**FIGURE 1:**
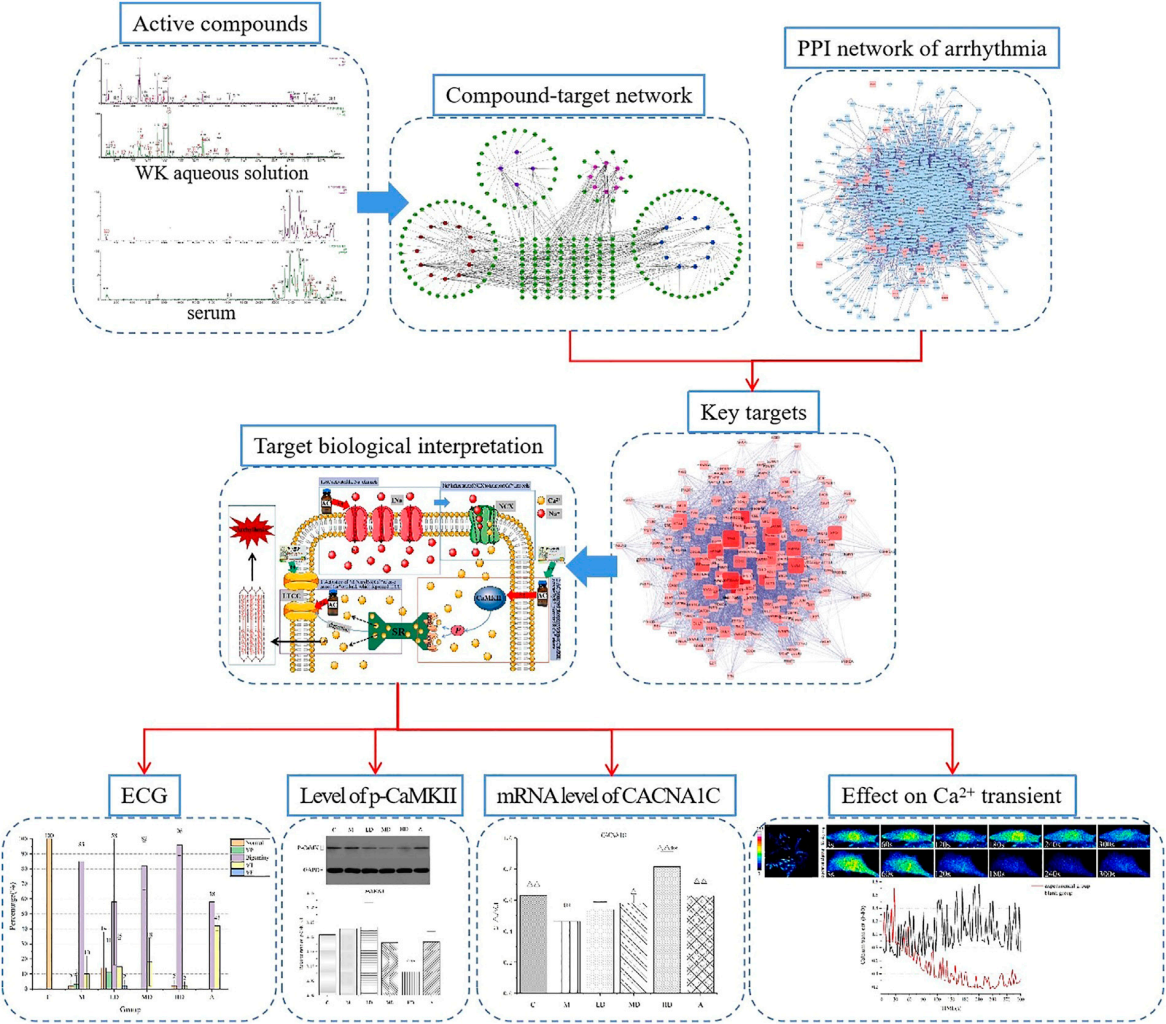
Flowchart for the systems pharmacology approach used in this study.

This study employed a systems pharmacology approach to investigate the pharmacological mechanisms of WK (Figure 1). The potential active compounds in WK were screened by ultra-performance liquid chromatography/quadrupole-time-of-flight mass spectrometry (UPLC/Q-TOF-MS), and their potential related targets were subsequently predicted using the weighted ensemble similarity method. The obtained targets were mapped onto relevant databases to detect their corresponding pathways. Subsequent experiments were further conducted to confirm whether the hypothetical results of the systemic pharmacology approach were correct (Huang et al., 2014; Li et al., 2014).

## Materials and Methods

### Wenxin Keli Component Identification and Prediction of Action Mechanism

#### Component Identification

The UPLC/Q-TOF-MS was employed to analyze aqueous solutions and serum components of WK qualitatively. The identified components were regarded as the potential active ingredients of WK.

### Target Prediction

Target Proteins of WK: TCM formulas can effectively prevent the devastating effects of complex diseases through the synergistic effects of multiple compounds and targets (Hao da and Xiao, 2014). Therefore, exploring the therapeutic targets of WK is required besides identifying its potential active compounds. In this study, an integrated *in silico* approach was employed to identify the target proteins for the possible active ingredients (components identified from 2.1.1) of WK. Predictive models including the TCMSP database (http://ibts.hkbu.edu.hk/LSP/tcmsp.php), STITCH (http://stitch.embl.de/) (Kuhn et al., 2012), Swiss Target Prediction (http://www.swisstargetprediction.ch/), Target-Prediction (http://prediction.charite.de/index.php?site=chemdoodle_search_target), and DrugBank (http://www.drugbank.ca/) were used to predict the target proteins of WK.

Target Proteins of Arrhythmia: Targets related to arrhythmia were obtained from “The gene connection for the heart” database (http//triad.fsm.it/cardmoc/).

### Network Construction

Compound-target Network: TCM formulas exert significant biological and pharmacological effects through multiple compounds and targets. This study constructed a compound-target (C-T) network based on the candidate compounds of WK and the potential targets to understand the complex interaction of compounds and their corresponding targets at a systems level.

Protein-protein Interaction (PPI) Network: Networks are used to view global relationships between nodes. In this study, a PPI network was used to identify potential target proteins associated with arrhythmia based on the arrhythmia targets identified in 2.1.2 using the Cytoscape 3.8.1 Bisogenet app (Smoot et al., 2011).

#### Identification of Key Targets

Experimental approaches for determining the targets of candidate drugs are costly, labor-intensive, and time-consuming. In this study, a systems pharmacology approach was used to determine the key targets through which WK exerts an antiarrhythmic effect. The Merge tool of the Cytoscape 3.8.1 app was employed to determine the common targets of the C-T network and PPI network. They were regarded as the key targets by WK in arrhythmia treatment.

#### Pathway Construction and Analysis

Signaling pathways are essential components of the system pharmacology that link receptor-ligand interactions to pharmacodynamics outputs (Iyengar et al., 2012). An incorporated “arrhythmia pathway” was established in this study to probe the action mechanisms of WK in arrhythmia treatment based on the current knowledge of arrhythmia pathology. The pathway information was acquired by inputting the key targets from 2.1.4 into the DAVID Bioinformatics Resources 6.8 (https://david.ncifcrf.gov/). An incorporated arrhythmia pathway was then assembled based on the basic pathway information by picking out the closely linked pathways related to arrhythmia pathology.

### Experimental Procedures

#### Source of Reagents and Antibodies

Acetonitrile (United States, 071757) and formic acid (analytical grade) were purchased from Fisher Scientific (United States, 154449). Purified water was acquired from a Milli-Q system (Millipore, Bedford, MA, United States). Medlab RU/4C50R multi-channel physiological signal acquisition and processing system were purchased from Shanghai Tongyu Teaching Instrument Manufacturing Co., Ltd (batch No. 17060155). WK was purchased from Shandong Buchang Pharmaceutical Co., Ltd, Tianjin, China (SFDA Approval number 1805106). Aconitine (AC) was sourced from Baoji Chenguang Biotechnology Co., Ltd., Shanxi, China (batch No. ha001198). Pentobarbital sodium was purchased from Baoji Chenguang Biotechnology Co., Ltd., Shanxi, China (batch No. B21882). In the same line, the p-CaMKII rabbit antibody was sourced from Abcam (ab32678), the GAPDH rabbit antibody from Hangzhou Xianzhi biology Co., Ltd, China (AB-P-R 001), and the HRP labeled Goat anti-rabbit second antibody from Wuhan bode Bioengineering Co., Ltd, China (BA1054). The cDNA synthesis kit (K1622) was purchased from ThermoFisher Scientific, United States.

### Sample Preparation

WK (5 g) was dissolved in 20.6 ml purified water (Nongfu Spring) and left to stand for 1 h. The solution was then filtered through four layers of gauze to filter out the dregs. The filtrate was subsequently centrifuged for 10 min at 10,000 rpm and 4°C, and 300 ml of the supernatant was stored at −20°C. The supernatant was diluted four times and filtered through a 0.22 μm filter membrane before injection for UPLC/Q-TOF-MS determination.

Blood samples (0.5 ml) were collected into serum tubes from the eye sockets of rats after 0.5, 1, 1.5, and 2 h of WK administration followed by immediate centrifugation at 3,500 rpm for 15 min at 4 C. The extracted serum was then subjected to UPLC/Q-TOF-MS determination.

#### Instrumentation and Chromatographic Conditions

Chromatographic separation was performed on an ACQUITY UPLC BEH C_18_ column (Φ 2.1 mm × 100 mm, 1.7 μm; Waters, United States) at a column temperature of 45°C and 5 μl injection volume. The sample was eluted at a flow rate of 0.3 ml min^−1^ in a gradient elution program of A (0.1% formic acid: water) and B (0.1% acetonitrile: water): 0–6 min (98–78% A); 6–10 min (78–70% A); 10–14 min (70–62% A); 14–18 min (62–62% A) 18–22 min (62–40% A); 22–26 min (40–0% A); 26–28 min (0–0% A); 28–29 min (0–98% A) 29–30 min (98–98% A). MS detection was performed on a high definition MS (HDMS) system (Waters, Te United States) with negative and positive electrospray (ESI) modes. Its sufficient sensitivity ensured that as many putative compounds as possible were identified. The optimized operating parameters were capillary voltage; 3.0 kV(Positive)/2.0 kV(Negative), source temperature; 120°C (Positive)/110°C (Negative), desolvation gas temperature; 450°C, desolvation gas (N_2_) flow rate; 800 L/h, collision energy (CE); 25 V, scanning time; 0.1 s, and scanning time interval; 0.02 s. Leucine-cerebral peptide solution (Waters) at a concentration of 200 pg/ml was used as the Lock-Spray calibration solution to ensure the accuracy and repeatability of the mass-to-charge ratio. The exact mass-to-charge ratios in positive and negative ion modes were [M+H]^−^ = 556.2771 and [M-H]^−^ = 554.2615, respectively. The data acquisition range and time were m/z 50–1000 and 0–30 min, respectively.

#### Animals Used and the Experimental Design

Clean grade male Sprague Dawley (SD, SPF level) rats aged 6–7 weeks and weighing 220 ± 20 g were obtained from the Experimental Animal Center of the Institute of health and environmental medicine, Academy of Military Medical Sciences, PLA, [license no. SYXK(Jin) 2014-0002]. All the animal-based experiments were approved by the Animal Care Committee of the Institute of Radiation Medicine of the Chinese Academy of Medical Sciences and performed following the relevant guidelines and regulations. Efforts were also made to minimize animal suffering. All rats were housed in the specific pathogen-free (SPF) animal laboratory of the experimental animal center of the Institute of Radiation Medicine Chinese Academy of Medical Sciences at a temperature of 23°C ± 2°C and 35% ± 5% relative humidity. The animals were acclimatized to the environment for 1 week with free access to a standard pellet diet before initiating the experiments. The rats were randomly divided into six groups, each comprising eight animals: the control (C) group, model (M) group, low-dose WK-treated (LD) group, medium-dose WK-treated (MD) group, high-dose WK-treated (HD) group, and the amiodarone (A) group. Intragastric administration of WK was once a day for 14 consecutive days in groups LD (2.41 g/kg/d), MD (4.82 g/kg/d), and HD (9.64 g/kg/d) (values are for the crude drug). Rats in group A were administrated with amiodarone (40 mg/kg), while those in groups C and M were administrated with an equal volume of distilled water.

#### Electrocardiogram Analysis

The rats were intraperitoneally anesthetized with 0.6% pentobarbital 30 mg/kg an hour after the last WK administration. They were then fixed on the plank in a prone position, followed by the insertion of electrodes into the subcutaneous tissue of the limb. The ECG parameters were subsequently recorded in each group using a Medlab RU/4C50R multi-channel physiological signal acquisition and processing system via a standard limb lead II. Rats in the other five groups, except those in group C, were subsequently injected with 0.001% AC 40 µg/kg after ECG stabilization to establish the arrhythmia model. The injection was through the caudal vein at a rate of 0.2 ml/min. Equal volumes of 0.9% normal saline were injected into rats in group C. The ECG of the rats was then observed in real-time for 20 min after the heart rhythm had normalized. The parameters measured included the initial time of arrhythmia and the types, occurrence, and duration of various VAs.

All animals were euthanized after ECG detection, and their hearts were immediately harvested and stored in liquid nitrogen awaiting WB and qRt-PCR analysis.

#### Western Blotting Analysis

The proteins were separated on a 10% SDS-PAGE and then transferred onto nitrocellulose membranes. The membranes were subsequently incubated with p-CaMKII rabbit antibody at 4°C overnight and then washed thrice with Tris-buffered saline (TBS) containing Tween 20 (TBST) to wash off the excess antibodies. The membranes were further incubated with horseradish peroxidase-conjugated secondary antibody for 2 h at room temperature. ECL visualization was then performed, followed by calculating the gray values using the Image J software (NIH Image, Bethesda, MD, United States). The intensity of the target proteins was normalized to that of an internal reference to determine their relative expression level.

#### Quantitative Real-Time PCR Analysis

The mRNA expression level of the predicated WK CAMKⅡ/CNCA1C/Ca^2+^ pathway was validated using Real-time Quantitative PCR (RT-qPCR) analysis. The total RNA of the myocardial pieces was first isolated using the Trizol reagent (Ambion, 15596-026), following the manufacturer’s instructions. The RNA samples (5 μg) were then reverse-transcribed to complementary DNA (cDNA) using a cDNA synthesis kit, followed by quantitative Rt-PCR on a 7500 Fast Real-Time PCR System (Applied Biosystems). The primer pair used was CAMKⅡ forward; 5′-CAA​GTT​CAT​CGA​GGT​CAC​CAC-3′ and reverse; 5′-ATA​CAC​AGC​TCT​CGT​CCT​CTG-3′, with RNA U6 as the internal control. The miR-1 specific primer sequences for quantitative real-time PCR were used for TaqMan MicroRNA Assays (Catalog number 4427975, Applied Biosystems). The expression levels of the miR-1 were subsequently normalized to U6 RNA expression levels and then calculated using the 2−ΔΔCt method (Livak and Schmittgen, 2001).

#### Laser-Scanning Confocal Calcium Imaging

Calcium imaging was carried out at room temperature within 6 h after isolation of cardiomyocytes from rats in the control group (Zhang et al., 2020). Ca^2+^ transients in the cardiomyocytes were recorded using an LSM-710 laser-scanning confocal microscope (Carl Zeiss, Inc, Germany) with a ×40 magnification, 1.3 numerical aperture oil immersion objective, and axial resolutions of 1.5 μm. Fluo-4 AM: Fluo-4AM+8μlDMSO+2μlF-127 was first prepared and then diluted 1,000 times using 1 ml DMem+1 μl Fluo-4. Cardiomyocytes were then incubated with 500–10,002 μl Fluo-4 AM (AAT Bioquest, Inc. Sunnyvale, CA, United States) for 20 min at 37°C and recorded in normal Tyrode’s solution. Fluo-4 was excited at 488nm, followed by measurement of the fluorescence emission at 505 nm. Images were acquired in the line-scan (X-T) mode with 512 pixels (pixel intervals 0.15 μm) per line at a rate of 3 ms per scan. The Ca^2+^ transients were then analyzed using a modified version of the MATLAB program, and their fluorescence emission intensity was expressed as F/F0, where F0 was the basal fluorescence intensity level. The recording was performed at 35°C.

#### Statistical Analysis

Data were expressed as the means ± SEM. Differences between the two groups were analyzed using the Student’s two-tailed *t*-test, while those between multiple groups were analyzed using one-way analysis of variance (SPSS, Inc., Chicago, IL, United States). *p* < 0.05 indicated significant differences between groups.

## Results

### Screening of Potential Active Compounds

**FIGURE 2:**
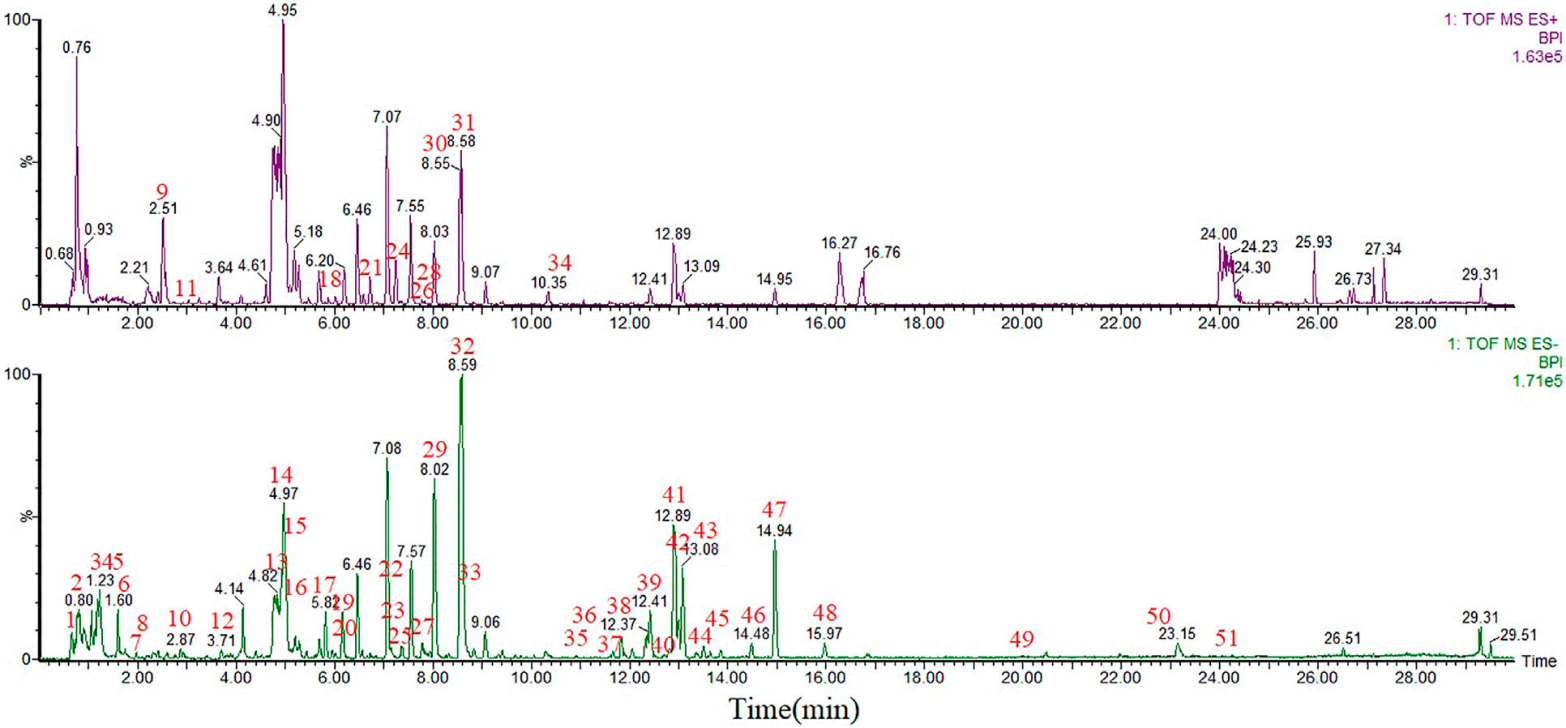
UPLC-MS base peak intensity chromatograms of WK aqueous solution in negative and positive modes.

**FIGURE 3:**
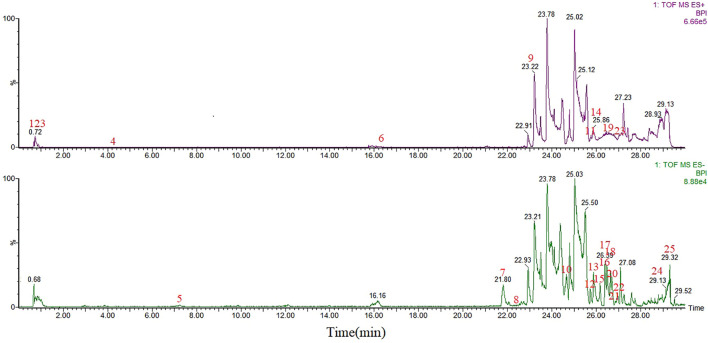
UPLC-MS base peak intensity chromatograms of serum after WK administration in negative and positive modes.

UPLC/Q-TOF-MS detected 51 components from the WK aqueous solution based on the m/z of precursors and fragments. Sugars and saponins were the main constituents of the components. The serum obtained after WK treatment contained 25 compounds, mainly composed of sugars, saponins, flavonoids, and amino acids. There were eight common components between the two solutions, and other 68 components were identified *in vitro* and *in vivo,* which were regarded as the potential active components in this study (Tables 1 and 2; Figures 2, 3).

**TABLE 1:** Main components of WK aqueous solution.

No.	tR/min	Nane	Formula	Calculated mass	Measured mass	Error (ppm)	MS/MS fragmentation	Herb
1	0.74	GUP	C6H12O6	180.156	180.063	6.201117318	179.0567[M-H]- 225.0627[M+CHOO]-	1,2,3,4
2	0.77	D-(+)-Xylose	C5H10O5	150.13	150.053	7.692307692	195.0520[M+CHOO]-	3
3	1.18	Jatamansinol	C14H14O4	246.262	246.089	9.931271478	291.0898[M+CHOO]-	3
4	1.28	Oroselol	C14H12O4	244.246	244.074	6.049382716	243.0643[M-H]-	3
5	1.46	Succinic acid	C4H6O4	118.088	118.027	6.923076923	117.0196[M-H]- 99.0118[M-H-H2O]- 73.0313[M-H-CO2]-	1,4
6	1.75	Leucine	C6H13NO2	131.175	131.095	9.615384615	130.0881[M-H]-	4
7	1.97	n-Butyl-β-D-fructopyranoside	C10H20O6	236.264	236.126	9.928825623	281.1265[M+CHOO]- 341.1112[M+2CHOO+H20-3H]-	4
8	2.03	Salicylic acid	C7H6O3	138.122	138.032	5.255474453	137.0246[M-H]-	4
9	2.51	Codonopsine	C14H21NO4	267.325	267.147	3.246268657	268.1541[M+H]+	1
10	2.85	5-Methoxymethyl furfural	C7H8O3	140.138	140.047	8.957055215	163.0386[M+Na]+	1
11	2.97	α-Copaene	C15H24	204.357	204.188	4.36123348	249.1600[M+CHOO]- 227.1767[M+Na]+	2
12	3.88	Chlorogenic acid	C16H18O9	354.311	354.095	4.532577904	353.0889[M-H]-	3
13	4.95	*Cryptotanshinone ①*	C19H20O3	296.366	296.141	2.033898305	295.1335[M-H]-	3
14	4.97	Tanshinone IIA	C19H18O3	294.35	294.126	6.962457338	293.1158[M-H]- 295.1329[M+H]+	3
15	5.03	*Tangshenoside III ②*	C34H46O17	726.725	726.274	4.409857328	771.2679[M+CHOO]-	1
16	5.07	(+)-Pinoresinol-O-β-D-glucopyranosyl(1→6)-β-D-glucopyranoside	C32H42O16	682.672	682.247	1.600587371	681.2385[M-H]-	4
17	5.82	3,5-Dimethoxyacetophenone	C10H12O3	180.203	180.079	7.29281768	181.0852[M-H]-	2
18	5.98	(1R)-2,3,4,9-tetrahydro-1H-$b-carboline-1-carboxylic acid	C12H12N2O2	216.24	216.09	5.760368664	217.0965[M+H]+	1
19	6.14	Heptanoic acid	C7H14O2	130.187	130.099	4.057142857	175.0978[M+CHOO]-	1
20	6.16	Hexyl-β-D-glucopyranosyl-(1→2)-β-D-glucopyranoside	C18H34O11	426.459	426.21	3.529411765	425.2039[M-H]-	1
21	6.72	Kingianoside A	C39H60O14	752.895	752.398	0.836653386	753.407[M+H]+	4
22	7.15	Azelaic acid	C9H16O4	188.223	188.105	3.262032086	187.0977[M-H]- 125.0979[M-H-H2O-CO2]-	1
23	7.19	20-O-Glucopyranosyl ginsenoside Rf	C48H82O19	963.165	962.545	0.793650794	1007.5438[M+CHOO]- 961[M-H]-	2
24	7.33	Myristicin	C11H12O3	192.214	192.079	0.932642487	193.0867[M+H]+	1
25	7.36	*Notoginsenoside Fe ③*	C47H80O17	917.14	916.54	1.746361746	961.5392[M+CHOO]-	2
26	7.65	Gynunol	C11H14O3	194.23	194.094	2.510460251	239.0926[M+Na]+	2
27	7.78	Notoginsenoside R3	C48H82O19	963.165	962.545	0.793650794	1007.5439[M+CHOO]-	2
28	7.83	Lobetyolin	C20H28O8	396.436	396.178		419.1693[M+Na]+	1
29	8.02	Notoginsenoside R1	C47H80O18	933.139	932.534	0.27607362	977.5327[M+CHOO]- 931.5277[M-H]-	1,2
30	8.56	ginsenoside Rh2	C36H62O8	622.884	622.444	8.780096308	623.4471[M+H}+	2
31	8.58	a-Curcumene	C15H22	202.341	202.172	5.960591133	203.1813[M+H}+	1
32	8.59	Ginsenoside Rg1	C42H72O14	801.024	800.492	0.059171598	845.4901[M+CHOO]- 799.4858 [M-H]-	2
33	8.65	Dioscin	C45H72O16	869.055	868.482	2.595837897	913.4776[M+CHOO]-	4
34	10.52	*Panaxytriol ④*	C17H26O3	278.392	278.188	5.519713262	279.1946[M+H]+	2
35	10.92	Notoginsenoside R6	C48H82O19	963.165	962.545	0.793650794	1007.5438[M+CHOO]- 843.4697[M+CHOO-Rha]-	2
36	11.3	Notoginsenoside T	C64H108O31	1373.537	1372.687	1.909620991	1371.6774[M-H]-	2
37	11.82	Notoginsenoside Fa	C59H100O27	1241.422	1240.645	2.532258064	1239.6346[M-H]- 1285.6412[M+CHOO]-	2
38	12.32	Notoginsenoside R4	C59H100O27	1241.422	1240.645	3.177419355	1239.6338[M-H]- 1285.6387[M+CHOO]-	2
39	12.42	Majonoside R1	C42H72O15	817.023	816.487	0.392638037	815.4799[M-H]- 861.4844[M+CHOO]-	2
40	12.51	Dehydroadynerigenin digitaloside	C30H42O8	530.658	530.288	4.365217391	575.2883[M+CHOO]-	4
41	12.9	Ginsenoside Rb1	C54H92O23	1109.307	1108.603	0.23465704	1107.5952[M-H]-	2
42	12.99	Ginsenoside Rg3	C42H72O13	785.025	784.497	0.434258142	829.4956[M+CHOO]- 783.4899[M-H]-	2
43	13.08	Ginsenoside Rh1	C36H62O9	638.883	638.439	1.171303075	683.4381[M+CHOO]-	2
44	13.38	Notoginsenoside Fc	C58H98O26	1211.396	1210.635	2.537190083	1209.6241[M-H]-	2
45	13.85	Ginsenoside Rb2	C53H90O22	1079.281	1078.592	0.204626335	1123.5906[M+CHOO]- 1077.5852[M-H]-	2
46	14.48	*Sanchinoside B1 ⑤*	C36H62O9	638.883	638.439	1.171303075	683.4393[M+CHOO]-	2
47	14.94	Ginsenoside Rd	C48H82O18	947.166	946.55	1.199596774	991.5469[M+CHOO]- 945.5406[M-H]-	2
48	15.97	Ginsenoside Re	C48H82O18	947.166	946.55	1.199596774	991.5469[M+CHOO]- 945.5410[M-H]-	2
49	20.01	*Ginsenoside Rh4 ⑥*	C36H60O8	620.868	620.429	3.654135338	665.4254[M+CHOO]-	2
50	22.58	*Atractylenolide II ⑦*	C15H20O2	232.323	232.146	1.335740072	277.1437[M+CHOO]-	1
51	24.14	*Ditertbutyl phthalate ⑧*	C16H22O4	278.348	278.152	4.418604651	277.1437[M-H]- 323.1353[M+CHO2]-	2

a 1: *Codonopsis pilosula* (Franch.) Nannf.; 2: *Panax notoginseng* (Burkill) F.H.Chen; 3: *Nardostachys jatamansi* (D.Don) DC.; 4: *Polygonatum kingianum* Collett & Hemsl. The eight components in italic are common components *in vivo* and *in vitro*. Reference: Wang and Qian (2010).

**TABLE 2:** Main components of serum after treatment of WK.

No.	tR/min	Nane	Formula	Calculated mass	Measured mass	Error (ppm)	MS/MS fragmentation	Herb
1	0.84	FOA	C5H4O3	112.084	112.016	0.888888889	135.0057[M+Na]+	1
2	0.85	Debilone	C15H22O2	234.339	234.162	1.361867704	257.1522[M+Na]+	3
3	1.03	Ricinin	C8H8N2O2	164.164	164.059	1.636363636	165.0667[M+H]+	1
4	4.31	Oroxin A	C21H20O10	432.381	432.106	3.76443418	433.1119[M+H]+	4
5	7.08	Picein	C14H18O7	298.291	298.105	7.138047138	297.0996[M-H]-	2
6	16.26	Isophytol	C20H40O	296.539	296.308	3.510971787	319.299[M+Na]+	3
7	21.79	*Panaxytriol ④*	C17H26O3	278.392	278.188	3.176895307	277.1796[M-H]-	2
8	22.4	*Atractylenolide II ⑦*	C15H20O2	232.323	232.146	0.83032491	277.1443[M+CHOO]-	1
9	23.21	Majonoside R2	C41H70O14	786.997	786.477	3.226205191	809.464[M+Na]+	2
10	24.6	*Ditertbutyl phthalate ⑧*	C16H22O4	278.348	278.152	0.431893688	277.1443[M-H]- 301.1418[M+Na]+	2
11	25.79	1-Methyl-4-isoallyl-cyclohexane	C10H18O2	126.111	126.032	4.152046784	171.1393[M+H]+	2
12	25.86	*Notoginsenoside Fe ③*	C47H80O17	917.14	916.54	2.68558952	915.5345[M-H]-	2
13	25.89	Linoleic acid	C18H32O2	280.452	280.24	5.197132616	279.234[M-H]-	2
14	25.97	*Tangshenoside III ②*	C34H46O17	726.725	726.274	2.035763411	727.28[M+H]+	1
15	26.19	5-Mpe-bis(hobz)phenol	C29H26O4	438.523	438.183	7.101449275	483.1843[M+CHOO]-	1
16	26.26	*Cryptotanshinone ①*	C19H20O3	C19H20O3	296.366	1.700879765	341.1384[M+CHOO]-	3
17	26.36	Notoginsenoside T1	C36H60O10	652.866	652.419	4.65437788	651.4141[M-H]-	2
18	26.41	Hexadecanoic acid	C16H32O2	256.43	256.24	7.254901961	255.2344[M-H]-	1,2,3
19	26.5	Protopanaxadiol	C30H52O3	460.743	460.392	3.436853002	483.38[M+Na]+	2
20	26.77	Lutein	C40H56O2	568.886	568.428	4.323001631	613.4233[M+CHOO]-	2
21	26.86	*Ginsenoside Rh4 ⑥*	C36H60O8	620.868	620.429	0.080775444	619.4213[M-H]- 665.4235[M+CHOO]-	2
22	26.98	*Sanchinoside B1 ⑤*	C36H62O9	638.883	638.439	3.328100471	637.4297[M-H]- 683.4306[M+CHOO]-	2
23	27.04	Dicaffeoyl phthalate	C24H38O4	390.564	390.277	5.907928389	391.2827[M+H]+ 413.2671[M+Na]+	1,2
24	29.2	Gypenoside XVII	C48H82O18	947.166	946.55	2.237687366	933.5447[M-H]-	2
25	29.3	Gypenosideix	C47H80O17	917.14	916.54	4.901960784	917.5432[M+H]+	2

a 1: *Codonopsis pilosula* (Franch.) Nannf.; 2: *Panax notoginseng* (Burkill) F.H.Chen; 3: *Nardostachys jatamansi* (D.Don) DC.; 4: *Polygonatum kingianum* Collett & Hemsl. The eight components in italic are common components *in vivo* and *in vitro*. Reference: Wang and Qian (2010).

### Target Prediction

Target proteins of WK: There were 192 potential targets that were predicted for the 68 candidate compounds. However, 34 candidate compounds had no corresponding targets based on this method (Supplementary Appendix S1). The five herbs had significant target overlap among them despite the differences in the number of each herb-related target. These results suggested that the different herbal drugs contained in WK possibly worked synergistically in regulating similar targets.

Target Proteins of Arrhythmia: The gene connection for the heart (http//triad.fsm.it/cardmoc/) revealed 61 **t**argets related to arrhythmia (Supplementary Appendix S2).

### Network Construction

Compound-target network: The C-T network embodied 226 nodes (34 candidate compounds and 192 potential targets) and 429 compound-target interactions. The mean degree value (the number of targets associated with it) of the candidate compounds was 41. Twelve compounds possessed a degree larger than 14 (2-fold degree median), indicating that they regulated multiple targets to exert varying therapeutic effects. Specially, five compounds, including Atractylenolide II, Tanshinone IIA, ginsenoside Rh2, Ginsenoside Rg1, and Jatamansinol, acted on 28, 24, 21, 20, and 16 targets, respectively. The compounds were regarded as the crucial active compounds for WK because of their prime positions in the network.

PPI network of arrhythmia: There were 1395 targets closely related to known arrhythmia targets. They were regarded to be indirectly related to arrhythmia.

### Identification of Key Targets

Analysis of the common targets of WK and arrhythmia revealed 33 key targets (Table 3). These targets were regarded to be the key targets for WK-mediated treatment of arrhythmia.

**TABLE 3:** Anti-arrhythmic targets of WK (gene name).

No.	Key target	No.	Key target	No.	Key target	No.	Key target	No.	Key target	No.	Key target
1	ADRA1D	7	BCL2L1	13	HSP90AB1	19	NFKB1	25	PLG	31	SOD1
2	ADRA2A	8	CASP3	14	JUN	20	NOS1	26	PRKACA	32	TNF
3	ADRB1	9	CDKN1A	15	KCNH2	21	NOS2	27	PTEN	33	TP53
4	AHSA1	10	EGFR	16	LYZ	22	NR3C1	28	RELA		
5	AR	11	ESR1	17	MDH2	23	OPRD1	29	SCN5A		
6	BAX	12	FOS	18	MYC	24	PFKM	30	SLC6A3		

### Pathway Construction and Analysis

**FIGURE 4:**
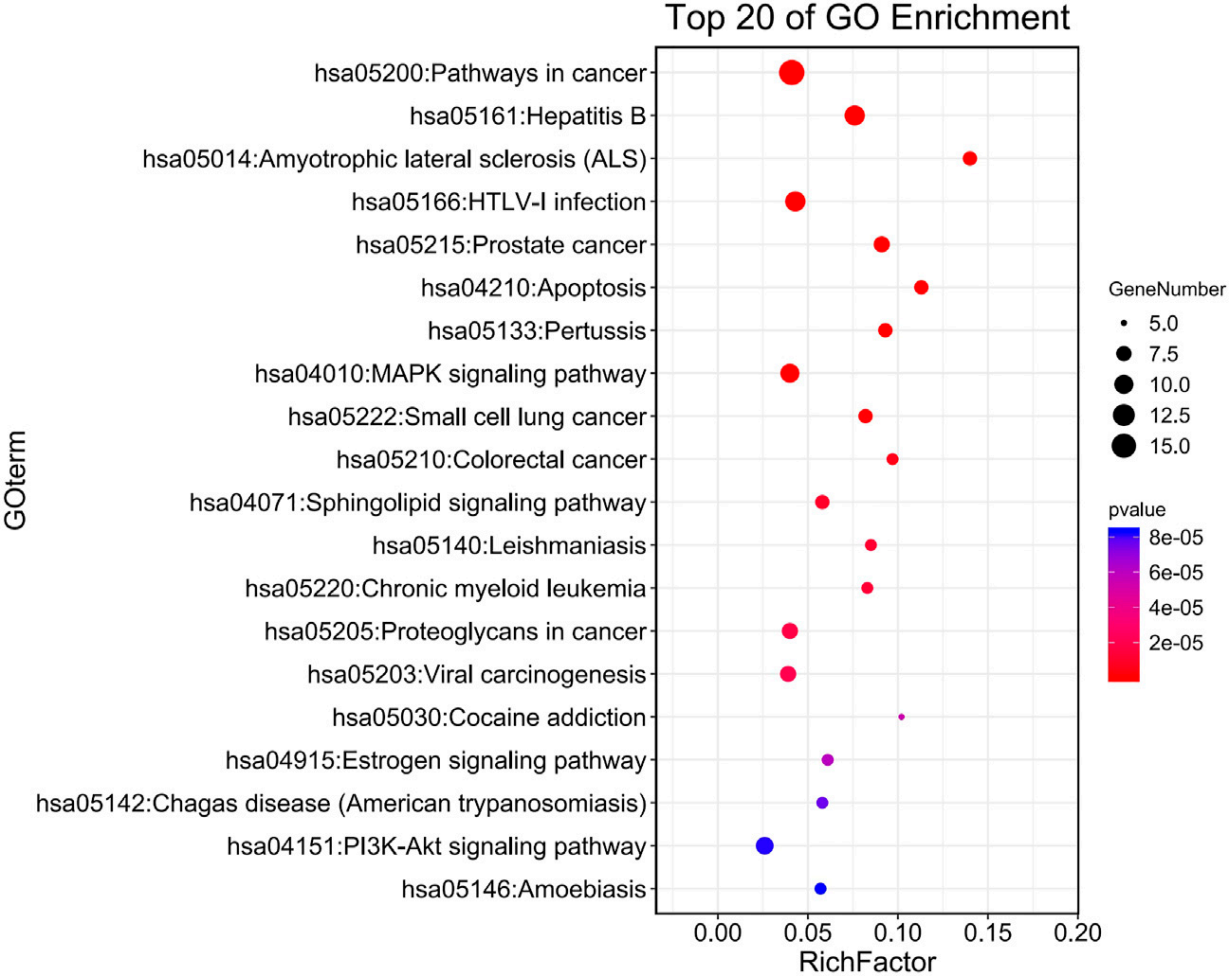
KEGG pathway analysis. The larger RichFactor is, the greater the degree of enrichment is; The range of *p* value is [0,1], and the closer it is to 0, the more significant the enrichment is.

The pathway information of the 33 key targets revealed 75 KEGG pathways, including the calcium, oxytocin, adrenergic signaling in cardiomyocytes, adipocytokine, and dilated cardiomyopathy signaling pathways. The pathways were closely associated with arrhythmias (Supplementary Appendix S3) based on their *p*-values. Figure 4 highlights the first 20 pathways obtained by DAVID enrichment. In addition, the calcium signaling pathway was involved in 34 pathways and indirectly related to 33 pathways. Therefore, we presumed that the calcium signaling pathway is closely related to arrhythmia. The CaMKⅡ, CNCA1C, and Ca^2+^ of the calcium signaling pathway were thus selected as the observation index in this study based on these findings combined with other relevant literature.

Calcium regulation plays a central role in cardiac function. The precise handling of cytoplasmic calcium concentration during the excitation-contraction coupling (ECC) is an essential aspect of arrhythmia pathophysiology. The L-type Ca^2+^ current (LTCC) plays a crucial role in the ECC. In the same line, membrane depolarization in ventricular myocytes during the action potential (AP) causes synchronous activation of multiple LTCCs, consequently triggering the release of Ca^2+^ from the sarcoplasmic reticulum (SR). These occurrences increase the intracellular Ca^2+^ that initiates contraction. Ca^2+^ influx via the LTCC provides a multifunctional signal that triggers muscle contraction, controls AP, and regulates gene expression (Shaw and Colecraft, 2013). As such, a dysregulation of the calcium channel function may lead to cardiac rhythm disorders. Cav1.2 is encoded by the Voltage-dependent L-type calcium channel subunit alpha-1C (CACNA1C) and regulated by the calcium/calmodulin-dependent protein kinaseII (CaMKII). Thus, an increase in the expression and activation of CaMKII causes an increase in CaV1.2 phosphorylation and diastolic SR Ca^2+^ leakage (Ai et al., 2005). The diastolic SR Ca^2+^ leak in return initiates early afterdepolarizations (EADs) (Wu et al., 1999) and delayed afterdepolarizations (DADs) (Xing et al., 2013).

Therefore, we hypothesized that WK could inhibit arrhythmias by regulating the CaMKII/CACNA1C/Ca^2+^ pathway. The assumption is that WK suppresses CaMKII phosphorylation and CACNA1C activation and antagonizes the intracellular Ca^2+^ overload to avoid the occurrence of EADs and DADs. CaMKII, CACNA1C, and Ca^2+^ concentration were thus selected as the observation indices of this study.

### Wenxin Keli Significantly Reduced AC-Induced Arrhythmia

**FIGURE 5:**
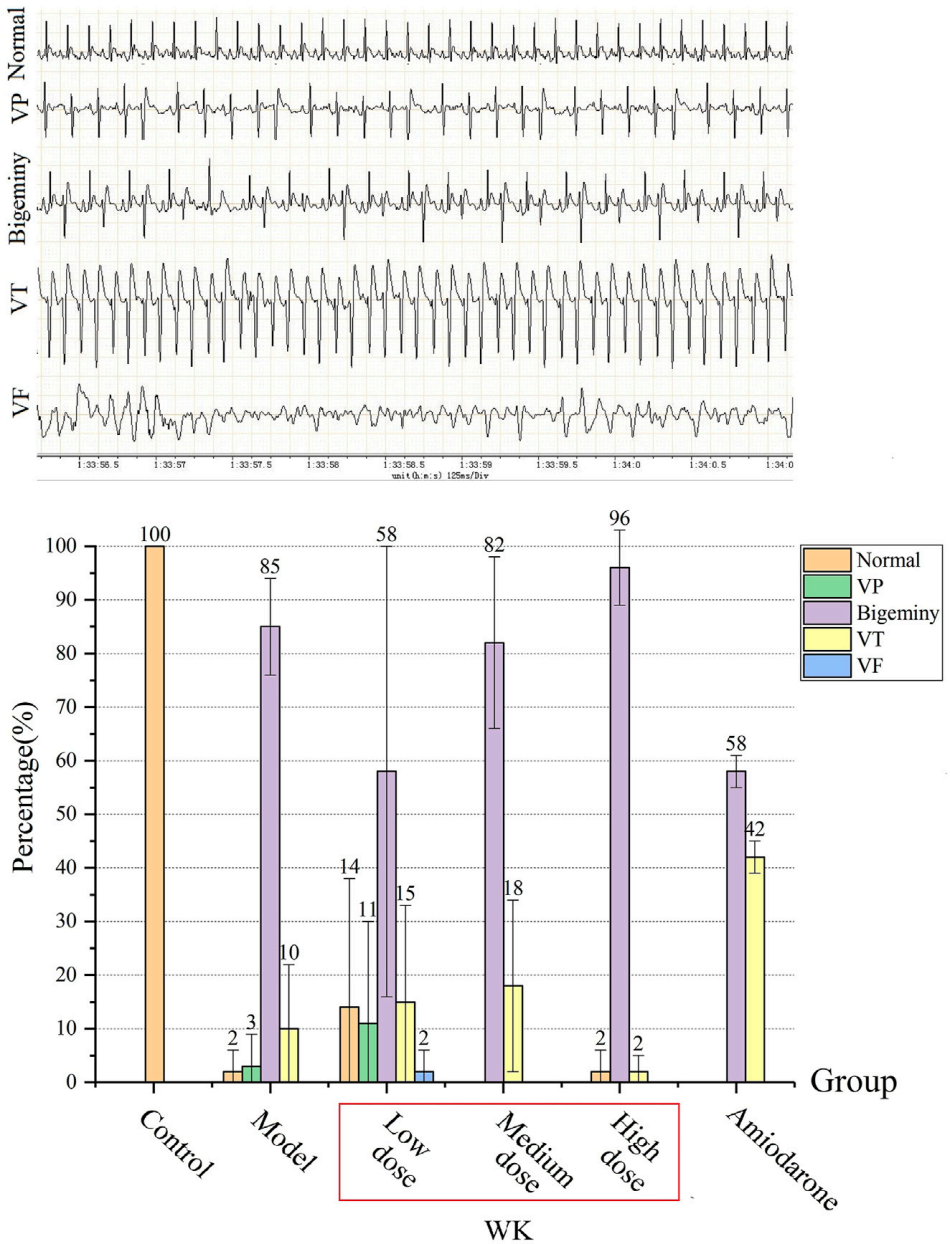
The percentage of arrhythmias’ duration (%) ($\bar{x}\pm s$, n = 8). VP: ventricular premature, VT: ventricular tachycardia, VF: ventricular fibrillation.

1) Mortality: There were no deaths in groups C, HD, and A during the experiments. However, two animals in group M and one animal in the groups LD and MD died. The animals died of ventricular fibrillation (VF) after pleomorphic ventricular tachycardia (Polymorphic VT). Nonetheless, the ECG recording time of the dead rats was very short and thus could not be compared with that of other rats. As such, we only analyzed the ECG of the surviving rats. All groups had different degrees of arrhythmia except group C.
2) The initial time and duration of arrhythmias: There were insignificant differences in the initial time and duration of arrhythmias among the five experimental groups (*p* > 0.05; Table 4). The average initial time and duration were 615.26 and 3,901.07 s, respectively. However, there were significant differences in arrhythmia percentage between the groups (*p* < 0.01; Figure 5). The arrhythmia order was ventricular premature (VP), bigeminy, VT, and VF based on severity. Notably, rats in group HD were mainly characterized by bigeminy (96%), with low proportions of VT and no VP during the onset of arrhythmia. The group exhibited the best inhibitory effect on tachycardia.
3) Types of arrhythmias: VT and bigeminy were observed in the five experimental groups. However, VP was observed in groups M, LD, and A, normal ECG was observed in groups M, LD, and HD, while VF was observed in group LD (Figure 5).
4) ECG parameters: Compared with ECG parameters before the experiment, R-R, P-R, and Q-T interval did not change in groups C and M. However, they were prolonged in groups LD, MD, and A, and shortened in group HD (Table 5). In the same line, APD was lengthened in groups LD, MD, and A, especially group Abut shortened in group HD. These findings suggested that high-dose WK played an antiarrhythmic role by shortening APD.

**TABLE 4:** The initial time and duration of arrhythmias ($\bar{x}\pm s$, *n* = 8).

Group	Control	Model	WK			Amiodarone
			Low dose	Medium dose	High dose	
Initial time(s)	None	534.67 ± 69.95	569.33 ± 40.61	696 ± 351.24	687 ± 109.16	589.33 ± 296.38
Duration(s)						
Total	1200	2,834.67 ± 420.39	2,619 ± 337.76	6,459 ± 5,264.6	2,342 ± 230.65	4,506 ± 791.57
Bigeminy	0	2,227.5 ± 507.01	1,583 ± 828.46	5,071.33 ± 3,571.4	2,301.67 ± 235.92	3,133.75 ± 1146.31
VT	0	413 ± 383.25	732.5 ± 406.59	2,081.5 ± 2,069.7	121 ± 0	1,929.33 ± 462.48
VP	0	346	1450	0	0	254
VF	0	0	193	0	0	0
Normal	1,200	964 ± 1,028.13	1827	0	170	0

VT, ventricular tachycardia; VP, ventricular premature; VF, ventricular fibrillation.

**TABLE 5:** ECG parameters under the influence of WK ($\bar{x}\pm s$, *n* = 8).

Group	Before			After		
	RR (ms)	PR (ms)	QT (ms)	RR (ms)	PR (ms)	QT (ms)
Control	132 ± 4.47	43.4 ± 1.34	55.2 ± 1.48	132 ± 4.47	43.4 ± 1.34	55.2 ± 1.48
Model	132 ± 4.47	45 ± 1.41	55 ± 1	132 ± 4.47	45 ± 1.41	55 ± 1
WK						
Low dose	128 ± 4.47	44 ± 1.58	55 ± 1	138 ± 4.47**	49.2 ± 1.3**	60.4 ± 0.89**
Medium dose	132 ± 4.47	44.6 ± 1.52	54.6 ± 1.95	142 ± 4.47**	50.2 ± 1.3**	61 ± 1.58**
High dose	134 ± 5.48	44.2 ± 1.92	53.6 ± 2.79	124 ± 5.48*	39.4 ± 1.95**	49 ± 2.55*
Amiodarone	132 ± 4.47	45 ± 2	54.2 ± 1.3	162 ± 4.47**	60 ± 1.58**	70 ± 1.58**

*p < 0.05, **p < 0.01, versus before experiment.

### Wenxin Keli Significantly Inhibits p-CaMKⅡ

**FIGURE 6:**
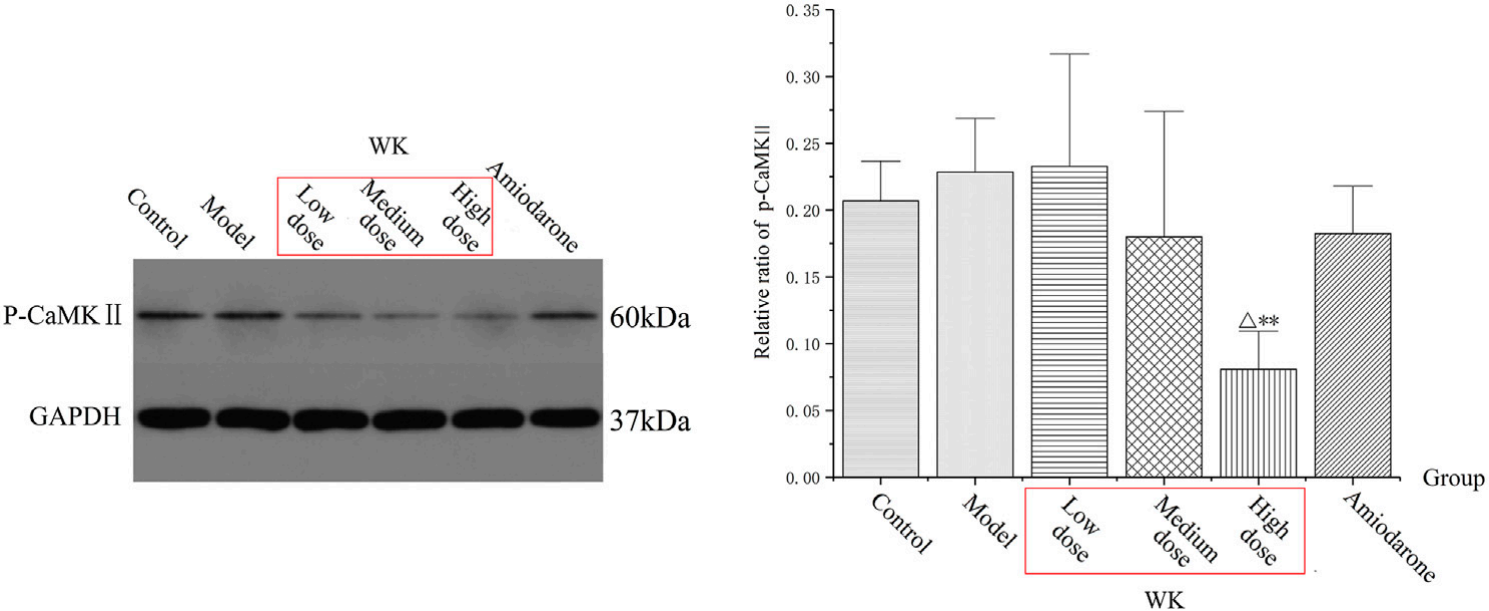
The levels of p-CaMKII protein. ($\bar{x}\pm s$, *n* = 8) *p < 0.05, **p < 0.01, versus the control group. ^△^ *p* < 0.05, ^△△^ *p* < 0.01, versus the model group.

Group M had increased p-CaMKⅡ levels than group C, suggesting that AC could activate CaMKII to induce arrhythmia. In the same line, the expression of p-CaMKII decreased in all groups except group LD after WK and amiodarone treatment, with group HD having the most significant decrease (*p* < 0.05). Notably, group HD had a significantly lower level of p-CaMKII than group C (*p* < 0.01), suggesting that a high WK dose markedly inhibited the expression of CaMKII (Table 6; Figure 6).

**TABLE 6:** The level of P-CaMK II protein ($\bar{x}\pm s$, *n* = 8).

Group	p-CaMK II
Control	0.21 ± 0.03
Model	0.23 ± 0.04
WK	
Low dose	0.23 ± 0.08
Medium dose	0.18 ± 0.09
High dose	0.08 ± 0.03^△△^**
Amiodarone	0.18 ± 0.04

*p < 0.05, **p < 0.01, versus the control group. ^△^ *p* < 0.05, ^△△^ *p* < 0.01, versus the model group.

### Wenxin Keli Significantly Increased CACNA1C

**FIGURE 7:**
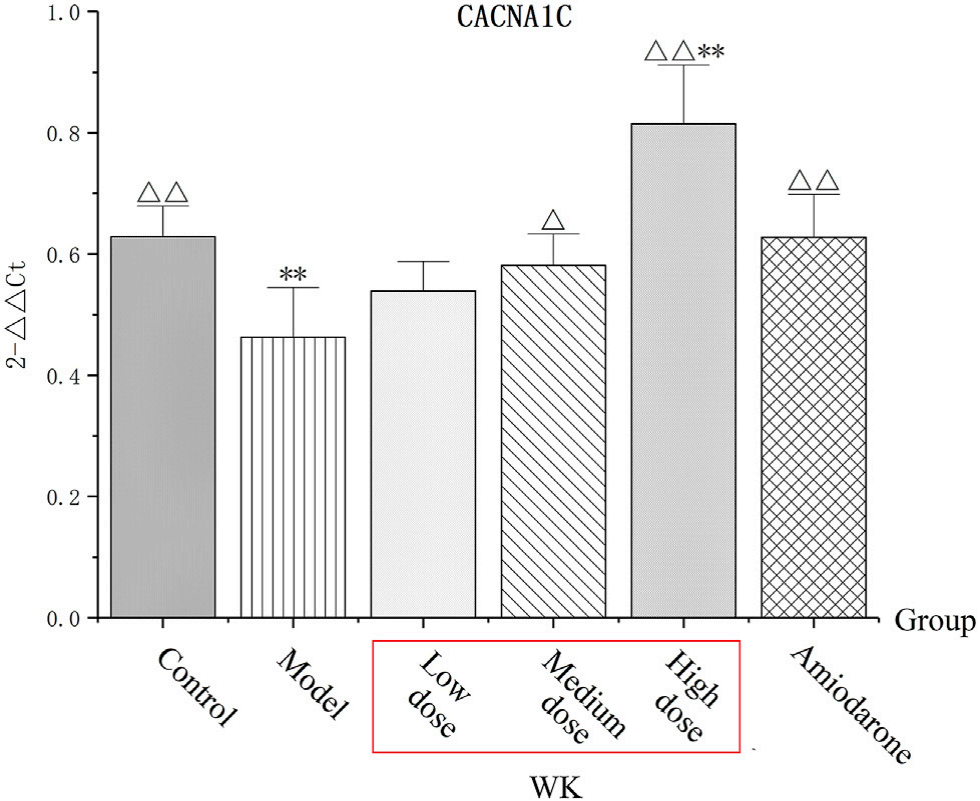
The level of CACNA1C. ($\bar{x}\pm s$, *n* = 8) *p < 0.05, **p < 0.01, versus the control group. ^△^ *p* < 0.05, ^△△^ *p* < 0.01, versus the model group.

Group M had decreased CACNA1C levels (*p* < 0.01) than group C, suggesting that AC could inhibit CACNA1C to induce arrhythmia. Compared with group M, the expression of CACNA1C increased in all groups except group LD after treatment of WK and amiodarone, with group HD exhibiting the most significant increase (*p* < 0.01). Notably, group HD had a significantly higher level of CACNA1C than group C (*p* < 0.01), suggesting that a high WK dose markedly increased the expression of CACNA1C (Table 7; Figure 7).

**TABLE 7:** The level of CACNA1C ($\bar{x}\pm s$, *n* = 8).

Group	CACNA1C
Control	0.63 ± 0.05^△△^
Model	0.46 ± 0.08**
WK	
Low dose	0.54 ± 0.05
Medium dose	0.58 ± 0.05^△^
High dose	0.81 ± 0.1^△△^**
Amiodarone	0.63 ± 0.07^△△^

*p < 0.05, **p < 0.01, versus the control group. ^△^ *p* < 0.05, ^△△^ *p* < 0.01, versus the model group.

### Wenxin Keli Significantly Reduced the Concentration of Intracellular Ca^2+^

**FIGURE 8:**
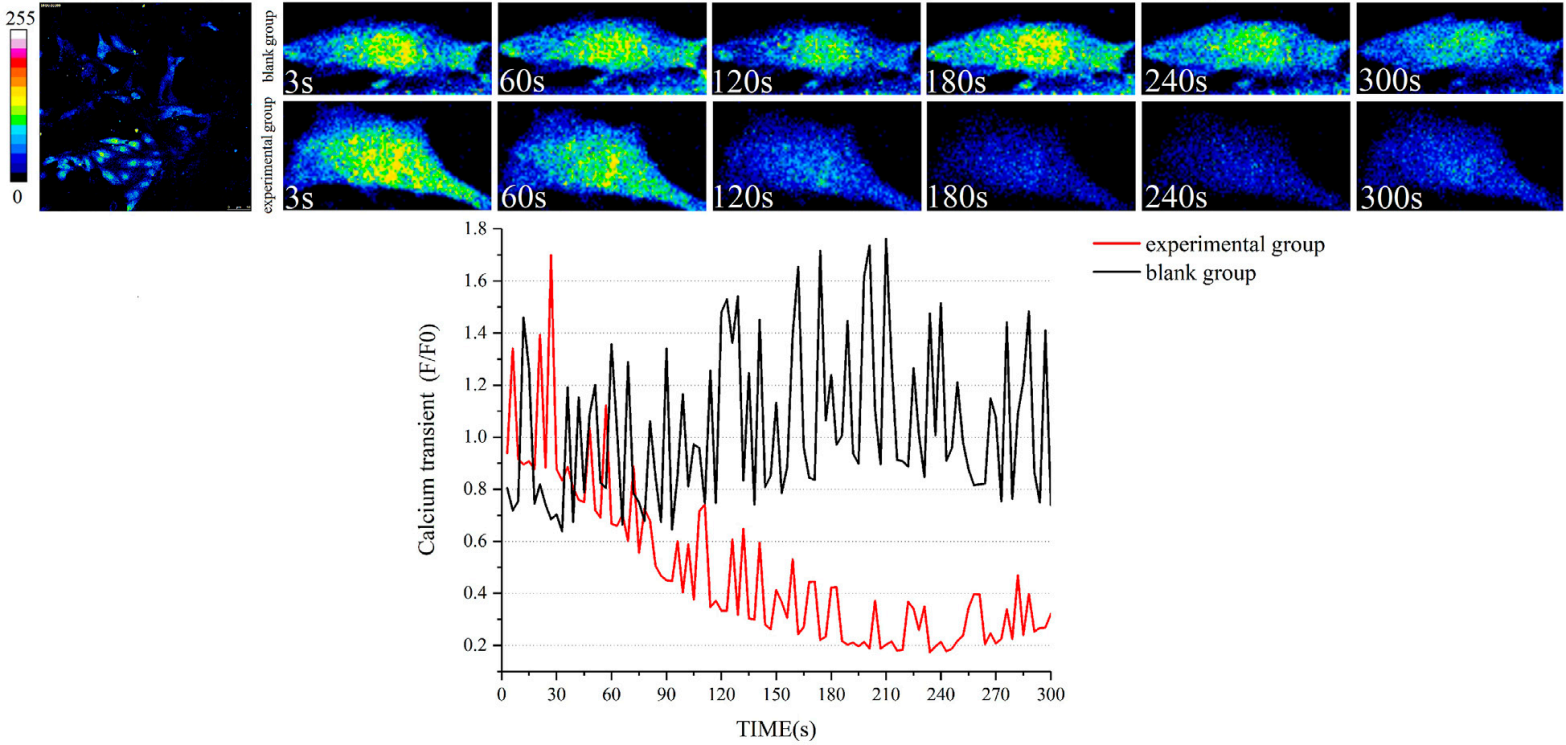
Effect of WK on cytoplasmic Ca^2+^ transient in cardiomyocytes. Black line: Ca^2+^ transient in the blank group; Red line: Ca^2+^ transient in the experimental group. *n* = 5 myocardial cells for each group. General Linear Model with Greenhouse-Geisser tests was applied for comparisons. The Ca^2+^ transient in the two groups was actually the same at baseline. However, after the infusion of high-dose WK, Ca^2+^ transient significantly reduced in the experimental group.

Cytoplasmic Ca^2+^ transients were determined in the experimental and blank groups (high dose of WK) to evaluate the time-course effects of WK on intracellular Ca^2+^ homeostasis. WK significantly reduced the resting Ca^2+^ ratio and the amplitude of Ca^2+^ ratio within 5 min after adding WK (*p* < 0.01). WK also induced a decrease in the cytoplasmic Ca^2+^ ratio within 5 min in a time-dependent manner (Figure 8; Supplementary Appendix S4). These results indicated that inhibition of Ca^2+^ overload is potentially an essential mechanism of the WK-induced antiarrhythmic effect.

## Discussion

### Systems Pharmacology Approach Used in Traditional Chinese Medicine Research

To date, the action mechanisms of TCM remain to be completely understood despite TCM having been known since ancient times to have therapeutic effects on human cardiovascular diseases (Jauhari et al., 2016). The drug discovery and development process are time-consuming, expensive, and challenging. The systems pharmacology approach can assist in shortening this time and potentially reduce the cost of drug research and development. In this paper, the pharmacology approach was used to predict the potential targets and action pathways of WK in arrhythmias treatment. Moreover, the study combined UPLC/Q-TOF-MS and animal-based experiments to identify WK’s active compounds and action mechanisms for arrhythmias treatment.

### Mechanism of AC-Induced Arrhythmias

AC is a major bioactive diterpenoid alkaloid derived from aconitum plants. It has been used in the past as an antipyretic, analgesic, antirheumatic drug, and a neurotransmission inhibitor (Herzog et al., 1964; Sato et al., 1979; Hikino et al., 1980; Fraser et al., 2003). However, it is toxic to the heart and the central nervous system (Honerjäger and Meissner, 1983; Catterall et al., 1992; Ameri, 1998). Arrhythmias are a major side effect of AC (Zhu et al., 2017), including the induction of VT, *torsades de pointes*, and VF (Lu and De Clerck, 1993). AC-induced arrhythmias are a consequence of a combination of multiple mechanisms. Studies postulate that AC increases the excitability of ectopic rhythms (Qiu et al., 2016), decreases the APA of papillary muscle APs, shortens the APD90 and APD30 (Chen et al., 2018), and leads to tachyarrhythmias.

1) Promote Na^+^ Influx: AC acts as an INa agonist that opens the Na^+^ channels during the depolarization/repolarization phases. This occurrence suppresses the conformational change of Na^+^ channels from the active to the inactive state, causing the membrane to remain depolarized (Wright, 2002; Wang and Wang, 2003). Large Na^+^ influx into the cytosol induces triggered activities (TA) (Sawanobori et al., 1987; Watano et al., 1999), thereby causing single or multifocal ectopic rhythms (Suzuki et al., 2014).
2) Lead to Ca^2+^ Overload: Abundant Na^+^ influx into the cytosol causes Ca^2+^ overload via sequential activation of electrogenic Na^+^-Ca^2+^ exchanger (NCX), thus inducing DAD and TA (Wang et al., 2008; Zhou et al., 2013). As a result, Ca^2+^ channel antagonists, such as verapamil, exhibit better therapeutic effects on AC-induced VTs than Na^+^ channel antagonists, such as quinidine (Takahara et al., 1999; Van Landeghem et al., 2007) in clinical practice.
3) Inhibition of Outward K^+^ Currents: AC significantly increases Ca^2+^ influx, inhibits outward K^+^ currents, prolongs repolarization, and produces DAD and reentry, resulting in arrhythmias (Mitamura et al., 2002).
4) Inhibition of LTCC: LTCC is activated upon membrane depolarization and Ca^2+^ influx. It triggers the release of Ca^2+^ via the Ca^2+^ release channels, ryanodine receptors (RyRs) of the SR. During normal AP, early LTCC peaks trigger robust SR release followed by partial inactivation because of two processes: Ca^2+^-and voltage-dependent inactivation. LTCC regulates AP repolarization and is vital in developing cardiac arrhythmias such as VF, the leading cause of sudden cardiac death (Moroz and Lipnitskii, 2006; Kranias and Bers, 2007). Studies postulate that AC-induced inhibitions of LTCC are vital in the proarrhythmic effects of AC in humans. AC can block LTCC following a mechanism independent from increased INa (Fu et al., 2007). The effects of AC on cardiac repolarization and beating frequency in hiPSC-CMs resemble that of nifedipine (Wu et al., 2017). Moreover, inactivating LTCC channels potentially contributes to intracellular Ca^2+^ elevation (Ferreira et al., 1997). In contrast, some studies suggest that AC accelerates the activation of LTCC and delays the inactivation of LTCC. These studies further suggest that AC increases Ca^2+^ influx through LTCC (Zhou et al., 2013). These data inconsistencies are attributed to the differences in the two types of myocytes because ionic channel characters of neonatal and adult ventricular myocytes are not completely identical. In this study, the expression of CACNA1C, the gene that encodes LTCC, was significantly inhibited by AC.
5) Others: AC also stimulates the vagus nerve and inhibits the sinoatrial node and conduction system, resulting in a slowed heart rate and conduction block (Jung et al., 2011; Ono et al., 2013). Moreover, the cardiac toxicity of AC is associated with an increase in free radicals caused by oxidative stress (Wang et al., 2008). AC cardiotoxicity is also attributed to Ca^2+^ overload and apoptosis via the p38 signaling pathway (Sun et al., 2014).

In the present study, the antagonistic effects of WK on AC-induced arrhythmia were observed in rats. ECG recording experiments demonstrated that AC administration decreased the heart rate and prolonged the Q-T intervals, leading to VP, coupled rhythm, and tachycardia. These occurrences ultimately induced VF and rat mortality. These findings are consistent with those of Qiu et al. (2016). In contrast, pre-administration of WK effectively delayed the onset of ventricular premature beat and coupled rhythm, thereby prolonging the survival time of rats.

### The Role of Ca^2+^ in Maintaining the Heart Rhythm

Cardiac contraction and relaxation are mediated by a precise and coordinated linkage of electrical activation (excitation) and intracellular Ca^2+^ homeostasis, resulting in excitation-contraction coupling. Ventricular AP starts with a sodium (Na^+^) influx through voltage-gated Na^+^ channels that depolarize the cell. Voltage-sensitive LTCC is activated at a certain threshold, allowing the influence of Ca^2+^ into the cytosol, which triggers a much larger Ca^2+^ release from the SR, the main intracellular Ca^2+^ storage organelle (Bers, 2002; Gambardella et al., 2018). Notably, sporadic subcellular localized Ca^2+^ releases are observed in myocardial cells during diastole as spatiotemporally restricted Ca^2+^ sparks (Cheng and Lederer, 2008). They originate from a single ryanodine receptor type2 (RyR2) or a RyR2s cluster and represent the elementary events of cardiac ECC. The recruitment and summation of many Ca^2+^ sparks at the beginning of systole produces abundant whole-cell Ca^2+^ transients. The process is referred to as Ca^2+^-induced Ca^2+^-release (CICR) and is the fundamental link between electrical and mechanical activation in the heart (Fabiato, 1983). The cytosolic Ca^2+^ then binds to troponin C and initiates myocardial contractions to form the cardiac systole. In contrast, myofilaments relax during diastole. The relaxation is caused by Ca^2+^ re-uptake into the SR by the SR Ca^2+^-ATPase type-2a (SERCA2a) that pumps Ca^2+^ back into the SR stores. It is also caused by Ca^2+^ extrusion and releases into the extracellular space through NCX, which exchanges three Na^+^ ions entering for one Ca^2+^ ion leaving the cell (Stern and Cheng, 2004). The NCX removes Ca^2+^ by generating a net inward depolarizing current, called the transient inward current (Iti). SERCA2a takes up approximately 63% of cytosolic Ca^2+^, while NCX extrudes 37% of Ca2+ in humans (Bassani et al., 1994; Terentyev et al., 2002). The depolarization and release of Ca^2+^ into the cytosol and its subsequent rapid re-uptake or extrusion results in a Ca^2+^ wave referred to as the Ca^2+^ transient. The amount of Ca^2+^ released from the SR directly correlates with the Ca^2+^ transient amplitude. It is responsible for the strength of systolic contraction. Notably, any alterations in intracellular Ca^2+^ handling results in electrical stability and cardiac contractility changes, leading to malignant ventricular arrhythmias. Principally, Ca^2+^ overload is an important AC mechanism that causes arrhythmia. Analyses of Ca^2+^-signaling parameters in cultured myocardial cells are traditionally based on the use of ratiometric Ca^2+^ dyes such as Fluo-4 followed by detection of spatiotemporal changes in their fluorescence intensities using epifluorescence or confocal microscopy (Whitaker, 2010; Prasad and Inesi, 2012). In this protocol, we describe an experimental approach suitable for evaluating Ca^2+^ signals in myocardial cells. This approach revealed that WK significantly reduces intracellular Ca^2+^ transients, thus highlighting it as a potential mechanism by which WK treats arrhythmias.

### The Role of Calcium/Calmodulin-Dependent Protein KinaseII in Antiarrhythmia

During the initial stage of AP, Ca^2+^ flowing into LTCC through mycoplasma voltage gating triggers SR to release large amounts of Ca^2+^. This myocardial contraction and blood drawing process driven by Ca^2+^ is called ECC (Winslow et al., 2016). In the same line, CaMKII is a key downstream regulator in Ca^2+^ related physiological activities, such as autophosphorylation and post-translational modification. It also plays an important role in the excitation-contraction coupling and relaxation events of cardiomyocytes (Jiang and Wang, 2020). CaMKII belongs to the subfamily of multifunctional Ser/Thr kinases, which phosphorylate various substrates and regulate numerous cellular functions (Soderling, 1999; Fujisawa, 2001; Hook and Means, 2001; Rusciano et al., 2012) intimately involved in heart diseases (Braun and Schulman, 1995; Erickson et al., 2008; Anderson et al., 2011). It catalyzes the phosphorylation of βThr498 (Grueter et al., 2006), additional sites on the CaV1.2 α-subunit (Lee et al., 2006; Hudmon et al., 2019), phospholamban (Bilezikjian et al., 1981), and ryanodine receptors (Wehrens et al., 2004). CaMKII mediated phosphorylation of the ryanodine receptor increases the open probability of sarcoplasmic Ca^2+^ load. In the same line, phosphorylation of the LTCC leads to a slower inactivation, while phosphorylation of phospholamban leads to an increase in sarcoplasmic Ca^2+^ load (Anderson, 2005; Guo and Duff, 2006). Consequently, these actions cause an up-regulated CaMKII activity during cardiac pathology, leading to an increased intracellular Ca^2+^ concentration. This increase leads to triggered activity via spontaneous diastolic Ca^2+^ release (Sossalla et al., 2010). Studies also postulate that acute CaMKII inhibition approaches are antiarrhythmic in numerous animal studies (Mazur et al., 1999; Gbadebo et al., 2002; Bourgonje et al., 2012; Driessen et al., 2014; Takanari et al., 2016; He et al., 2019). In this study, the level of p-CaMKII in the model group was higher than that of the control group, suggesting that AC activated CaMKII to induce arrhythmia. AC inhibits the expression of CACNA1C, thereby inhibiting Ca^2+^ from entering cardiomyocytes through LTCC. Moreover, SR Ca^2+^ release is essential in activating CaMKII, which subsequently induces phosphorylation of RyRs and promotes the release of Ca^2+^ from SR. Our data showed that WK can inhibit the expression of CaMKII, suggesting it was one of the mechanisms by which WK treats arrhythmias.

### Fascinating L-Type Ca^2+^ Current

The precise handling of cytoplasmic Ca^2+^ concentration during ECC is an important aspect of arrhythmia pathophysiology. ECC is an essential effector of the LTCC Cav1.2, the main isoform expressed in ventricular cardiomyocytes (Bers, 2002). The L-type refers to ‘‘long-lasting,’’ a historic nomenclature that antedates molecular identification of the determinants of ICa. ICa is a slow inward current that contrasts the more rapid kinetics of the Na^+^ current. Cav1.2 is a pore-forming a-subunit of LTCCs, indicating that it is a member of the voltage-gated (v) Ca^2+^ channel family. It is structurally homologous to voltage-gated Na^+^ channels and belongs to a family of six membrane-spanning voltage-gated ion channels. CACNA1C, CACNB2, and CACNA2D1 genes encode the pore-forming a-subunit (Cav1.2) and the b2 and a2d ancillary subunits that form the cardiac Ca^2+^ channel, which generates the LTCC (Rougier and Abriel, 2016). LTCCs are distributed in small clusters of about 10–12 channels along the sarcolemma of these cells (Bers and Stiffel, 1993; Franzini-Armstrong et al., 1999; Soeller et al., 2007; Dixon et al., 2015). LTCCs open to allow Ca^2+^ to enter the cell once the membrane potential is reached during the plateau phase of the ventricular AP. The Ca^2+^ signal is amplified via Ca^2+^-induced Ca^2+^ release by opening RyRs from the SR, causing a cell-wide increase of Ca^2+^, which triggers cell contraction (Cheng et al., 1993; Cheng et al., 1996). ICa is a critical determinant of intracellular Ca^2+^ transients that trigger transmitter release, secretion, and contraction (Catterall, 2000). The size of the intracellular Ca^2+^ transients in the heart is determined by the release of Ca^2+^ from intracellular stores and the size of the LTCC (Bers, 2002). LTCC is an essential source of inward current (ICa) for prolonging APD (Alseikhan et al., 2002). CaV1.2 proteins are subject to multiple modes of regulation that determine the number of available channels by trafficking or modulating channel activation or inactivation via protein kinases and accessory proteins. As such, numerous variables determine LTCC activity. The human CACNA1C gene located on chromosome 12 encodes for the pore-forming CaV1.2 subunit protein of the cardiac LTCC (Powers et al., 1991; Soldatov et al., 1998). Abnormal expression of CACNA1C thus leads to LTCC dysfunction.

ICaL must inhibit the transportation of Ca^2+^ out of the cytosol primarily via the sarcolemmal NCX and the SR Ca^2+^-ATPase (SERCA) for relaxation to occur in normal conditions. This phenomenon consequently takes Ca^2+^ back into the SR. NCX operates in both the Ca^2+^ efflux and influx (or reverse) modes, depending on the internal and external concentrations of Na^+^ and Ca^2+^. INa is elevated in AC-induced myocytes, while NCX works almost exclusively in the Na^+^ extrusion mode, thus significantly increasing the amount of Ca^2+^ influx. This influx raises the cellular and SR Ca^2+^ content, resulting in larger Ca^2+^ transients. In contrast, other studies postulate that SR Ca^2+^ release modulates the sarcolemmal ICaL, suggesting a retrograde in communication between the SR and the sarcolemmal LTCC in cardiac ECC (Balog and Gallant, 1999). Moreover, AC -induces SR Ca^2+^ leakage through the RyR2 channel, thereby depressing ICaL. These reports support the retrograde hypothesis in communication between the SR Ca^2+^ release channel and the LTCC (CACNA1C) (Fu et al., 2008). Notably, this communication may be mediated by a direct interaction between the two-channel proteins, LTCC and RyR2, in some cases (Nakai et al., 1996). Studies postulate that AC decreased the expression level of SERCA but increase that of NCX (Zhou et al., 2013).

This study showed that AC has a significant inhibitory effect on LTCC, indicating that AC-induced intracellular Ca^2+^ overload is not achieved by increasing LTCC. However, these findings contrasted those of Souza et al. (2019) and Koenig et al. (2014), who reported that AC increased LTCC expression. Notably, the studies report the existence of NCX and a retrograde in communication between the SR Ca^2+^ release channel and LTCC. In this study, WK significantly reduced intracellular Ca^2+^ transients, thus confirming that the increased expression of NCX and the presence of Ca^2+^ influx mode were the primary causes of AC-induced arrhythmia. AC also increased the expression of p-CaMKⅡ, thus promoting the release of Ca^2+^ from SR. The Ca^2+^ release of SR inhibited LTCC because of the retrograde communication between them. QRT-PCR results of CACNA1C also confirmed these findings. Similarly, laser-scanning confocal calcium imaging directly demonstrated the inhibitory effect of WK on intracellular Ca^2+^ concentration. Maintaining intracellular Ca^2+^ balance may thus be the primary mechanism of WK against arrhythmia.

### Highlights and Innovation of the Pharmacology Approach

1) It is a good model for TCM research: The approach also detected the chemical components of WK besides the anti-arrhythmia mechanism and correlated the potential active components with their pharmacodynamic mechanisms. The research model was more systematic and complete, thus proving a good model for TCM research.
2) It provides a research direction of antiarrhythmic effects of TCM: Besides the most relevant calcium signaling pathway, other signaling pathways were also detected, thus providing a good direction for subsequent studies. The pathways included the oxytocin signaling pathway, adrenergic signaling in cardiomyocytes, adipocytokine signaling pathway, and dilated cardiomyopathy associated with the metabolism of adrenaline, angiotensin, fats, glycometabolism, apoptotic factors, and other arrhythmia-related factors. These findings demonstrate that TCM treatment of diseases has a multi-target comprehensive effect, thus further proving that systems pharmacology is an effective method in TCM study.
3) Construction of appropriate disease model: The present study used AC to construct a pure arrhythmia rat model instead of the toxic arrhythmia that causes myocardial infarction and heart failure. The pure arrhythmia model was more conductive to reveal the pathogenesis of arrhythmia because it did not interfere with the complex pathological mechanisms.
4) New antiarrhythmic strategy: This study demonstrated that WK had therapeutic effects in AC-induced rat arrhythmias by inhibiting p-CaMKⅡ, over-expressing CNCA1C, and inhibiting intracellular Ca^2+^ transients. CaMKII and NCX play a vital role in Ca^2+^ balance. As such, blocking CaMKII and NCX can be a safe and effective strategy against arrhythmias.

## Conclusion

### Antiarrhythmic Potential Active Components and Targets of Wenxin Keli

UPLC/Q-TOF-MS identified 68 components both *in vivo* and *in vitro* as the potential active components of WK. Among them, 33 key targets were screened based on their corresponding targets of WK potential active ingredients and targets of arrhythmia. These targets were regarded as the antiarrhythmic targets of WK (Figure 1). This study proposes that the calcium signaling pathway is the primary signaling pathway targeted by WK in arrhythmia treatment because of the WK targets, including CaMKII, CNCA1C, and Ca^2+^, involved in the calcium signaling pathway. It supposes that WK inhibits arrhythmia by regulating CaMKII, CNCA1C, and intracellular Ca^2+^ transients.

### Mechanism of Treating Arrhythmia Using Wenxin Keli

**FIGURE 9:**
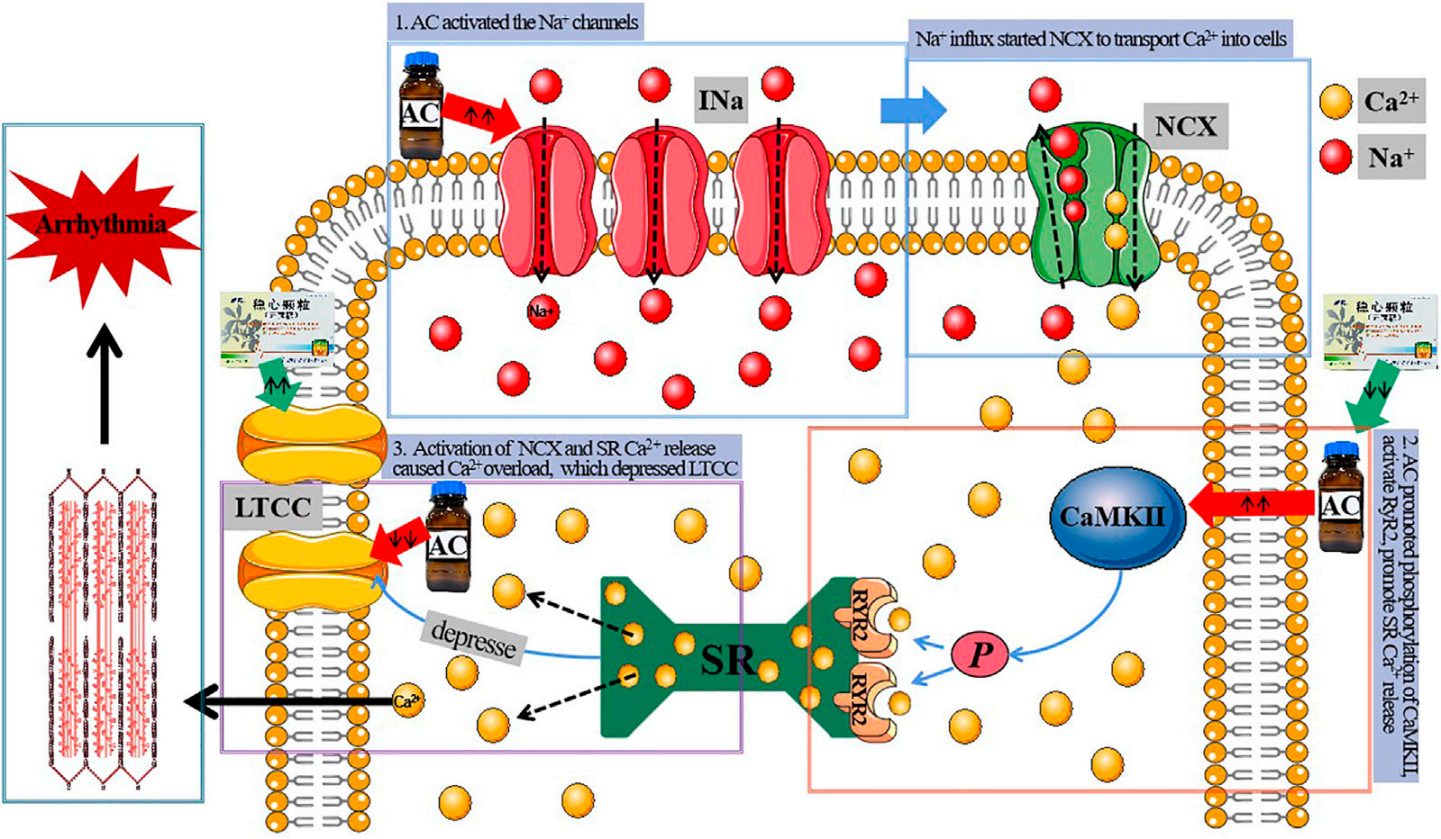
Mechanism of treating arrhythmia with WK.

Ca^2+^ is both a charge carrier and a second messenger. As such, slight alterations in Ca^2+^ concentration during ECC significantly impacts arrhythmia vulnerability (Eisner et al., 2017). AC activates the Na^+^ channels, thus causing a substantial Na^+^ influx into the cytosol, which induces TA. In the same line, NCX transport Ca^2+^ into cells to reduce the Na^+^ content. AC also promotes the phosphorylation of CaMKⅡ, activates RyR2, and promotes SR Ca^2+^ release. Intracellular Ca^2+^ markedly increases and induces Ca^2+^ overload, consequently increasing the frequency of propagating Ca^2+^ waves and subsequent escalation of the propensity for triggered arrhythmia (Tiso et al., 2001; Wang et al., 2008). The intracellular Ca^2+^ overload triggered by Ca^2+^ signals is a potential mechanism of AC-induced cardiac arrhythmia (Zhou et al., 2013). There is a retrograde communication between the SR Ca^2+^ release channel and the LTCC (CNCA1C). Moreover, AC-induced SR Ca^2+^ leakage through the RyR2 channel further inhibits LTCC (Figure 9).

However, pretreatment with WK lowers mortality, lessens malignant arrhythmias, and shortens RR, PR, and QT intervals than AC-induced rats. WK induces some antiarrhythmic effects by inhibiting p-CaMKⅡ and intracellular Ca^2+^ transients and overexpressing CNCA1C, thus suppressing SR Ca^2+^ release, which is the most probable mechanism of WK inhibition of the Ca^2+^ overload. Maintaining intracellular Ca^2+^ balance is, therefore, an essential mechanism of WK against arrhythmia. The antiarrhythmic mechanism of WK also suggests that TCM has a multi-target comprehensive effect against diseases. Notably, high dose WK has the best impact and should thus be highly considered during clinical treatment.

### Tips for Antiarrhythmic Targets

CaMKII activity reflects the frequency of cytosolic Ca^2+^ oscillations. As such, its activation is directly linked to increased APD, EADs, and arrhythmias (Mazur et al., 1999). The Ca^2+^ homeostatic proteins involved in E-C are CaMKII targets (Couchonnal and Anderson, 2008). APD prolongation because of increased ICaL requires SR Ca^2+^ to activate CaMKII, which then binds to and phosphorylates β2a to trigger EADs. Therefore, CaMKII can serve as a novel therapeutic target for arrhythmias.

Similarly, NCX blocking is a promising safe and effective strategy against repolarization-dependent arrhythmias because it causes intracellular Na^+^ accumulation and reversal, leading to a high cytoplasmic Ca^2+^ influx.

## Data Availability

The original contributions presented in the study are included in the article/Supplementary Material. Further inquiries can be directed to the corresponding authors.

## Abbreviations

TCM, traditional Chinese medicine; WK, Wenxin Keli; Ito, potassium channel current; INa, sodium current; ICaL, L-type calcium current; APD, action potential duration; AC, aconitine; ECG, electrocardiogram; qRt-PCR, quantitative Real-time PCR; ECC, excitation-contraction coupling; LTCC, L-type Ca^2+^ current; AP, action potential; SR, sarcoplasmic reticulum; CACNA1C, voltage-dependent L-type calcium channel subunit alpha-1C; CaMKII, calcium/calmodulin dependent protein kinaseII; EADs, early afterdepolarizations; DADs, delayed afterdepolarization; VF, ventricular fibrillation; VT, ventricular tachycardia; VP, ventricular premature; TA, triggered activities; NCX, Na^+^-Ca^2+^ exchanger; RyRs, ryanodine receptors; CICR, Ca^2+^-induced Ca^2+^-release; SERCA2a, Ca^2+^-ATPase type-2a.
