# Supplementary figures and images for: Wenxin Keli for the Treatment of Arrhythmia—Systems Pharmacology and In Vivo Pharmacological Assessment
Source: Front Pharmacol. 2021 Aug 26;12:704622. doi: 10.3389/fphar.2021.704622 (PMC8426352; doi:10.3389/fphar.2021.704622)

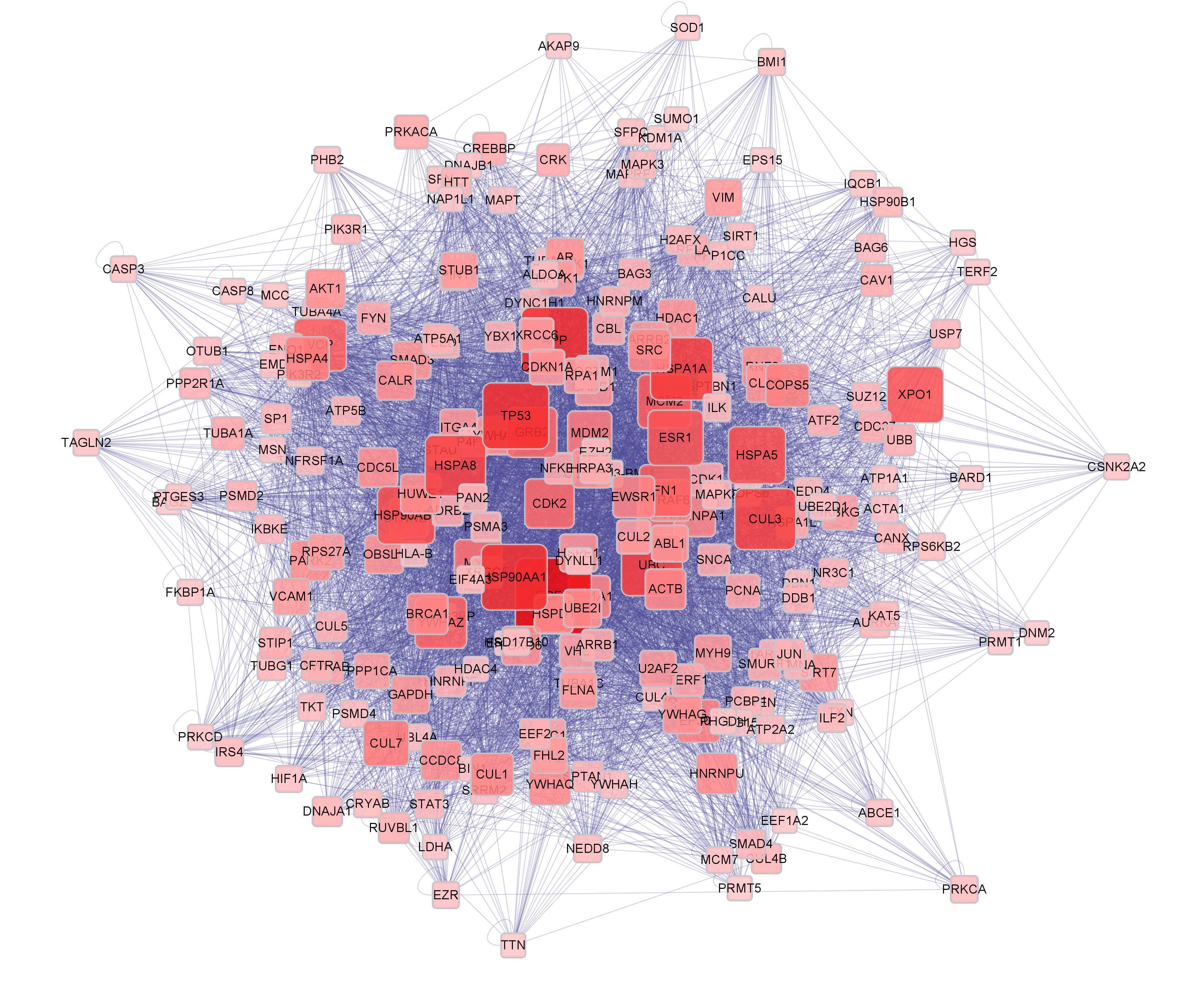

Supplement: Supplementary file 2 [file Image3.JPEG]

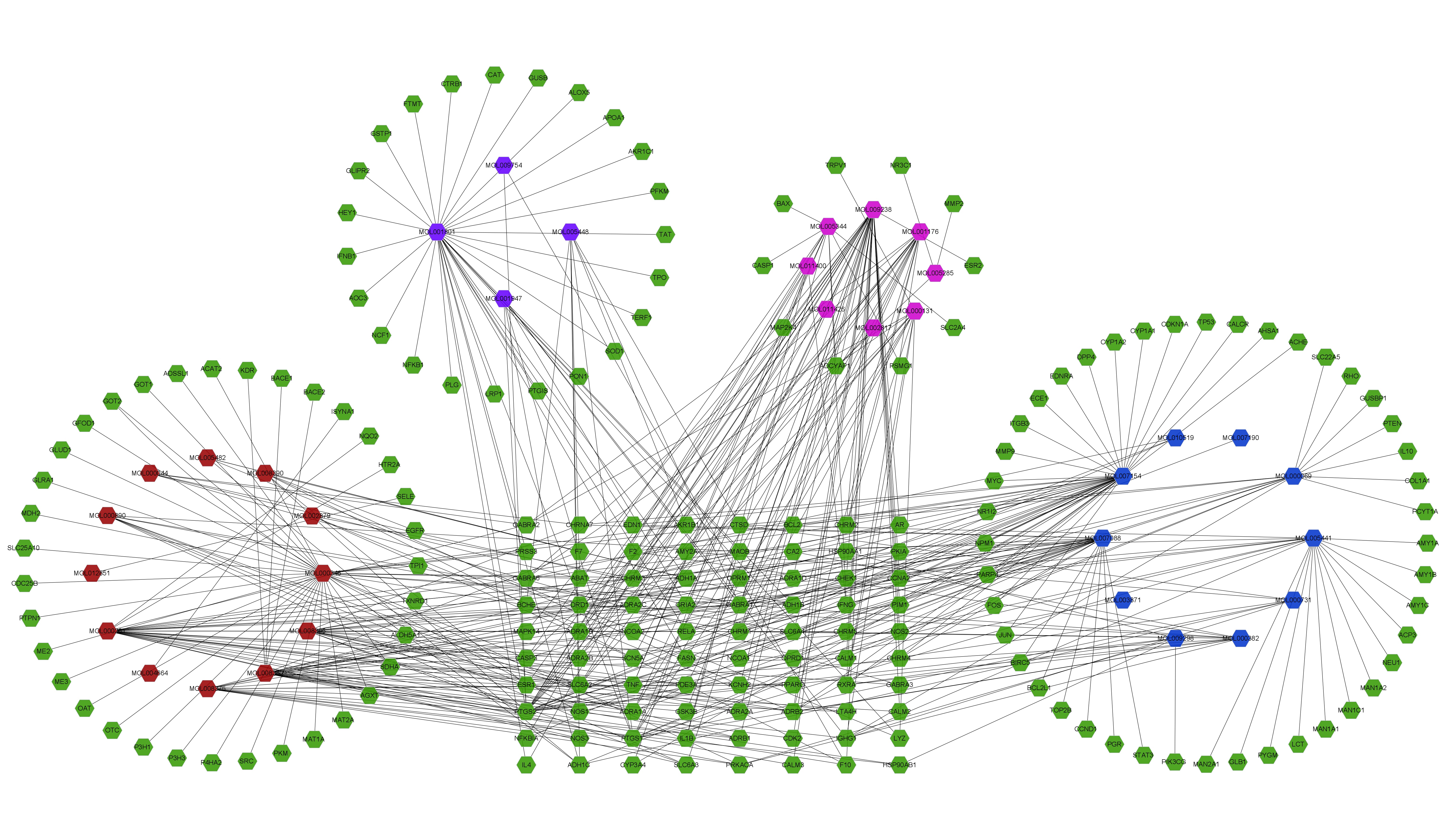

Supplement: Supplementary file 4 [file Image1.JPEG]

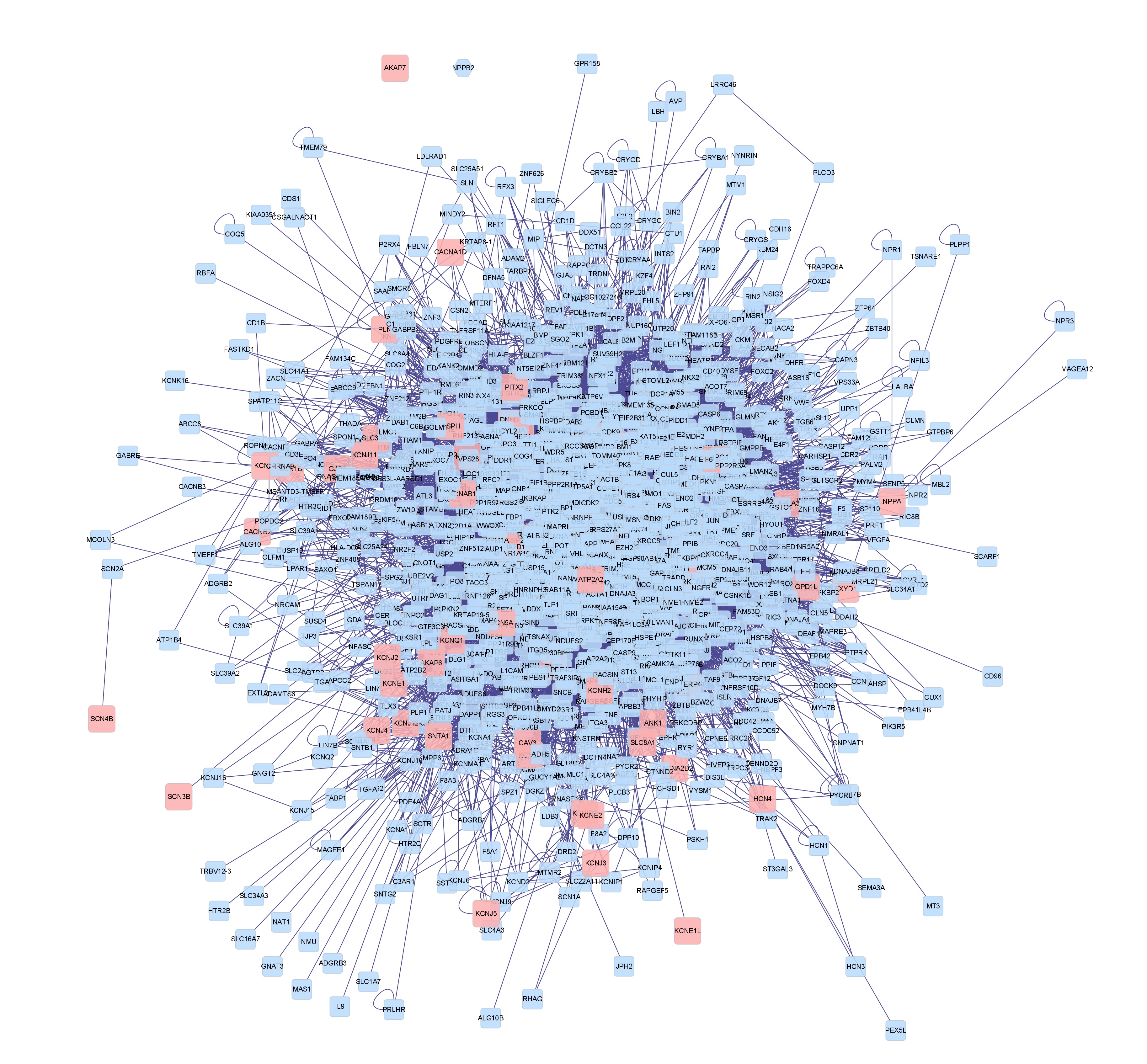

Supplement: Supplementary file 5 [file Image4.JPEG]

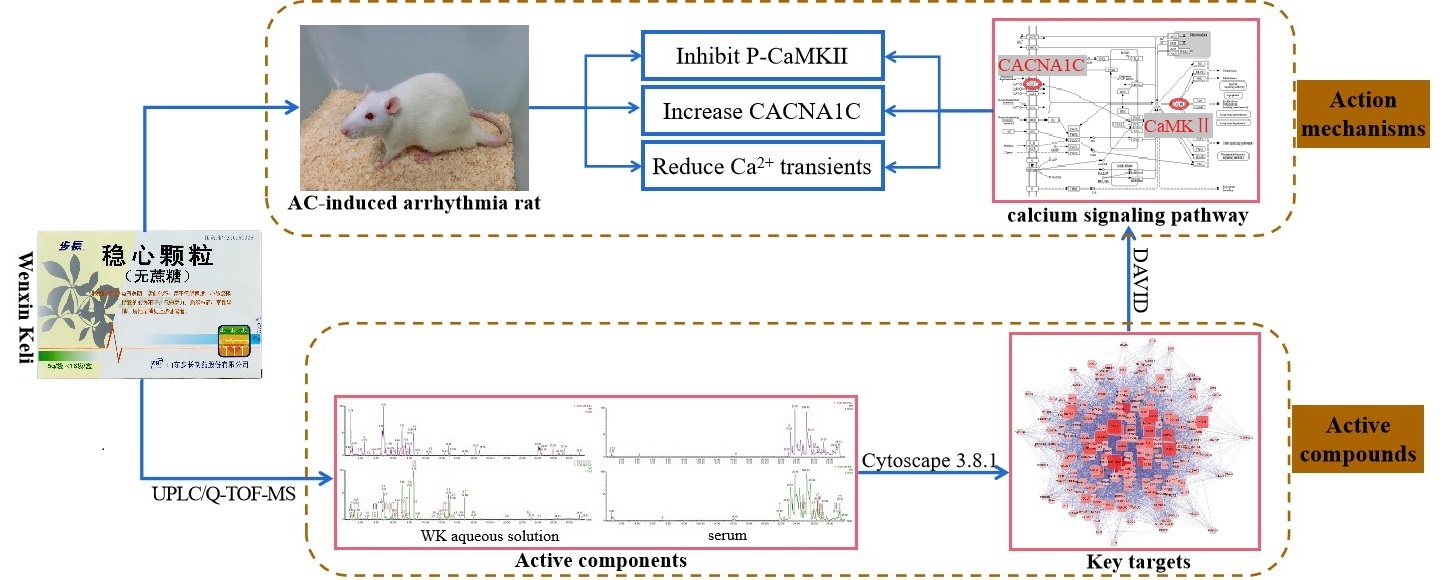

Supplement: Supplementary file 6 [file Image2.JPEG]
